# Heterotopic Ossification: Molecular Drivers, Subtype-Specific Mechanisms, and Translational Therapeutic Advances

**DOI:** 10.3390/biom16040585

**Published:** 2026-04-15

**Authors:** Sihong Chen, Hui Lin

**Affiliations:** 1 School of Basic Medical Sciences, Jiangxi Medical College, Nanchang University, Nanchang 330006, China; jp4217122055@qmul.ac.uk; 2 Queen Mary School, Jiangxi Medical College, Nanchang University, Nanchang 330006, China

**Keywords:** heterotopic ossification, fibrodysplasia ossificans progressiva, progressive osseous heteroplasia, signaling pathway, targeted therapeutics

## Abstract

Heterotopic ossification (HO), the pathological formation of mature bone in non-skeletal soft tissues (e.g., muscles, tendons), severely impairs patient mobility and quality of life. Despite decades of research, systematic analysis of signaling networks across HO subtypes (acquired traumatic HO, hereditary Fibrodysplasia Ossificans Progressiva (FOP), Progressive Osseous Heteroplasia (POH)) remains insufficient, and clinical therapies suffer from high recurrence and severe side effects. This review synthesizes recent advances in HO pathogenesis: FOP involves gain-of-function activin A receptor type I (ACVR1) mutations (mostly R206H), disrupting bone morphogenetic protein (BMP)/Activin A signaling; POH arises from paternal guanine nucleotide-binding protein, alpha-stimulating activity polypeptide (GNAS) loss-of-function mutations, derepressing Hedgehog signaling via reduced cyclic adenosine monophosphate (cAMP)/protein kinase A (PKA) activity; tHO features trauma-induced inflammation/hypoxia activating BMP/transforming growth factor–beta (TGF-β) pathways. Key signaling crosstalk (e.g., BMP-Yes-associated protein (YAP)-Indian hedgehog (IHH)) is integrated, and novel therapies (ACVR1 inhibitors, Activin A antibodies, retinoic acid receptor gamma (RARγ) agonists, adeno-associated virus (AAV)-mediated ACVR1 silencing) are highlighted, with emphasis on subtype-specific efficacy. A stratified, mechanism-based HO management framework is proposed, aiming to accelerate precision therapy development and advance understanding of aberrant tissue regeneration.

## 1. Introduction

Bone formation typically occurs only during prenatal development and postnatal skeletal repair. However, following severe trauma or specific genetic mutations after birth, bone can develop outside its usual anatomical and temporal contexts, a process known as heterotopic ossification (HO) [1]. HO characterizes the abnormal lamellar bone forms in soft tissues, including skeletal muscles and connective tissues [2]. This condition can disrupt normal physiological processes and physical movement, leading to long-term pain, joint stiffness, pressure-induced skin ulcers, venous thromboembolism, and other related issues [3,4]. Initial manifestations of HO may present as pain, erythema, oedema around the joints, and progressive reduction in joint mobility [5]. It significantly impacts patients’ daily activities, emotional well-being, and overall healthcare burden [6]. HO frequently arises as a complication in post-traumatic recovery contexts such as bone fractures, spinal cord trauma, brain injuries, explosion-related injuries, major burns, and surgical procedures, including hip arthroplasty, acetabular surgeries, and elbow surgeries [6,7,8,9]. Three critical conditions necessary for osteogenesis are typically absent in normal soft tissues: osteogenic precursor cells, osteogenic signal induction factors, and a suitable local microenvironment [10,11]. In addition, HO occurs in soft tissues, driven by the aberrant activation and osteogenic differentiation of a variety of tissue-resident progenitor and stem cells [12]. HO can develop via intramembranous and/or endochondral processes, akin to normal skeletal morphogenesis, highlighting the involvement of diverse cellular mechanisms, and the position of ossification occurs [13]. A critical feature of HO is the transformation of skeletal progenitor cells into chondrocytes and osteoblasts within soft tissues rather than happening within the skeletal structure.

HO is broadly categorized into hereditary and acquired types [14]:

Hereditary HO is divided based on distinctive mutations, including Fibrodysplasia Ossificans Progressiva (FOP) and Progressive Osseous Heteroplasia (POH). These conditions are driven by genetic alterations leading to progressive and sometimes widespread ectopic bone formation. Acquired HO, such as traumatic HO (tHO), typically arises following injury or trauma.

FOP, characterized by postnatal progressive ossification of soft tissues, typically begins in early childhood with the first HO episode occurring within the first ten years of life [15,16,17]. The global prevalence of FOP is estimated at 0.6–1.39 cases per million population, with no significant predilection for ethnicity, race, gender, or geographic bias [18]. Most individuals with FOP exhibit a congenital deformity of the great toe at birth, while displaying no other apparent abnormalities. Most children with FOP experience intermittent, painful inflammatory swellings in the soft tissues, referred to as “flareups” [19]. However, with age, the progressive accumulation of HO limits their range of motion, induces significant pain, and leads to gradual immobilization, ultimately impairing their quality of life [20]. Unlike traumatic HO, FOP flare-ups can be provoked by seemingly trivial injuries, including intramuscular injections, dental procedures, muscle overexertion, blunt injuries, or viral infections, leading to further HO progression. The principal genetic factor responsible for FOP is a recurrent heterozygous missense gain-of-function mutation in the ACVR1 gene, resulting in ~97% of FOP patients with an amino acid alteration at codon 206 (R206H) [15]. This mutation leads to dysregulated BMP signaling, both ligand-independent and ligand-stimulated [21], and disrupts signaling pathways across cells, tissues, and organ systems throughout life [22]. Surgical excision, typically used for traumatic HO, is contraindicated in FOP because of the progressive nature of the condition. Treatment for FOP includes glucocorticoids to manage inflammation, along with NSAIDs, radiation, and other modalities, each with its own advantages and drawbacks [19].

POH is another type of hereditary HO driven by an autosomal dominant mutation, primarily resulting from the functional inactivation of the paternal allele of the guanine nucleotide-binding-subunit (GNAS) gene, mainly occurring in deep connective tissues and skeletal muscles [23]. The HO in POH principally happens via intramembranous ossification [24]. However, based on transcriptome analysis, a study found that genes closely related to chondrogenesis and chondrocyte differentiation were highly expressed in POH samples [25], suggesting that the ossification process in POH may involve both endochondral and intramembranous ossification mechanisms [26]. The mainstream hypothesis for POH is that it is caused by a heterozygous inactivating mutation in the GNAS gene [25], with 60% to 70% of cases due to inactivating pathogenic variants of GNAS in paternal inheritance [27]. Additionally, there is another hypothesis suggesting that POH may originate from an early post-fertilization event, leading to loss of heterozygosity (LOH) at the GNAS locus, thereby triggering segmental biallelic mosaicism. However, it still needs some molecular support [28]. In POH patients, clinical presentations can vary significantly, even among individuals with identical mutations and genetic backgrounds [29]. Notably, certain patients without a GNAS mutation may still exhibit a familial background of HO and/or hormone resistance [30,31]. Therefore, POH is primarily influenced by genetic inheritance but is also affected by various other factors [23].

Situated on chromosome 20q13.3, the GNAS gene encodes the α subunit of the G-protein subunit and is involved in regulating the transcription of other imprinted genes [23]. This gene is critical in osteogenesis, and its mutation leads to disruptions in bone development and regulatory mechanisms. POH exhibits a specific pattern of HO, the maintenance of normal endocrine function, and accelerated calcification progression, distinguishing it from other diseases caused by GNAS gene inactivating mutations [32]. Clinically, POH can be classified into two types: one with widespread HO without causing functional impairment, and the other with progressive HO, which ultimately results in complete joint ankylosis [23]. In POH, the molecular microenvironment of the affected tissue is hypothesized to be a pivotal determinant of both disease initiation and advancement [25]. In recent years, new phenotypes of POH have been reported. Studies have shown that intravenous bisphosphonates, corticosteroids, and topical sodium thiosulfate failed to demonstrate clinical improvement. Surprisingly, surgical excision of calcified lesions combined with physical therapy led to significant improvement in the patients’ mobility [32].

In contrast, traumatic HO (tHO) typically arises after central nervous system damage or direct musculoskeletal trauma [33], which is controlled by mixed endochondral and intramembranous ossification [34]. Neurological heterotopic ossification (NHO), a significant complication of traumatic brain injury, is recognized as a more severe variant of HO, often resulting in considerable pain and marked mobility limitations compared to conventional HO cases [35]. Unlike genetic HO, which involves mutations, tHO results from trauma-induced factors, including inflammation and hypermetabolism, which disrupt the tissue repair cascade and lead to inappropriately activated osteogenic or osteochondrogenic mechanisms [36,37]. Epidemiologically, individuals with trauma-related HO are categorized into noncombatant civilians and military combatants [38]. Existing studies indicate that musculoskeletal injuries from military conflicts disrupt the normal healing process, resulting in abnormal scar tissue development that frequently includes ectopic bone formation and fibrotic changes [39].

This review mainly focuses on tHO, FOP, and POH, analyzing their associated signaling pathways to explore potential therapeutic targets.

## 2. Prevalence

According to the available data, HO development following trauma is a significant complication in 12% to 25% of fractures [40], and recent reports from the military suggest that up to 60% of blast injuries caused by trauma are associated with HO [41], which indicates a high risk. In terms of the anatomical sites of onset, HO predominantly manifests in the gluteus medius muscle following surgical trauma, such as total hip arthroplasty (THA), and the incidence of HO could reach around 40% [42]. Additionally, diagnosis of HO occurs in 2% to 90% of patients post-THA [43] and in 10% to 20% following brain and spinal cord injuries, resulting in joint pain and functional impairment [33]. Also, the frequency of HO at the elbow varies considerably. For instance, in cases of distal intra-articular humerus fractures, 35% of patients required surgical intervention for severe ossifications, whereas the same procedure was necessary in 26% of patients with elbow dislocation fractures [44]. HO is observed in 20% of forearm fractures and reaches a prevalence of 52% in femoral shaft fractures [33]. In patients with burns, the likelihood of HO significantly increases when the affected area exceeds 20% of the total body surface area [45], with severe burns associated with HO in 60% of cases [38]. HO may worsen in patients who do not receive adequate bed rest and immobilization following a fracture or joint surgery [42].

## 3. Pathogenesis of Heterotopic Ossification

### 3.1. Cellular Participants and Key Mechanisms in HO

A majority of non-hereditary traumatic HO and FOP both predominantly develop in soft connective tissues via intra-cartilaginous ossification, accompanied by a strong inflammatory reaction during the incipient periods of bone formation [46]. Its pathogenesis involves a variety of cell types and molecular pathways. Inflammatory cells, such as macrophages, mast cells, and lymphocytes, initiate the differentiation of precursor cells in both acquired and hereditary forms of HO [17,47]. These immune cells release a range of cytokines and growth factors, such as IL-1β, IL-6, OSM, NT-3, activin A, BMPs, TGF-β, and SP [17,47,48]. This differentiation process culminates in HO formation and is characterized by the upregulation of key osteogenic markers/proteins, including ALP, OCN, OPN/SSP1, and BSP [49].

Both mesenchymal stem cells (MSCs), which originated from bone marrow and local tissue, play pivotal roles in tissue improvement and revitalization. Their functions are mediated through precisely regulated interactions, which involve paracrine signaling and differentiation into osteoblasts, chondrocytes, fibroblasts, myoblasts, or adipocytes, influenced by microenvironmental cues [19]. Due to the heterogeneity of the etiology of ectopic ossification, several cell types contribute to ectopic bone formation, with the primary ones being hematopoietic cells, endothelial cells, fibro-adipogenic progenitors (FAPs), myosatellite cells, pericytes, Hoxa11+ mesenchymal stromal cells, tendon and ligament progenitor cells (such as Tendon-derived stem cells) [12]. Due to the widespread distribution of FAPs in various tissues and their established role in HO, FAPs may contribute to the development of most cases of HO. Hematopoietic-derived cells play a role in stimulating the osteogenic potential of these cells [12].

Generally, the pathological process of HO development involves a dual-phase mechanism: initial inflammation and connective tissue degradation (phase 1), followed by bone formation (phase 2) [46].

Inflammation shows a central involvement in the development and progression of HO. Tissue injury-induced acute inflammation not only initiates HO but also drives its early progression through the activation of the immune system [17,50,51,52]. It is worth noting that FOP can be subject to mild trauma and strong acute inflammatory responses triggered after vaccination, which is related to genetic variation in ectopic ossification [17,53]. Furthermore, inflammation not only contributes to the onset of HO but also influences its progression. Inflammatory mediators exhibit dual effects by simultaneously promoting bone formation and exacerbating inflammation. For instance, IL-6, a well-known proinflammatory cytokine, has been demonstrated to facilitate the conversion of MSCs into osteogenic progenitors, inhibit osteoblast apoptosis, and enhance angiogenesis in bone healing and remodeling processes [51]. Based on the central role of inflammation, targeted therapy targeting inflammatory cytokines and their signaling pathways has become a potential strategy for HO management.

The bone formation phase is categorized into three sequential phases: fibroproliferation coupled with angiogenesis, chondrogenesis, and osteogenesis [46,54]. Soft tissue injury triggers immune activation and local hypoxia, which act as predisposing factors. Within this specific microenvironment, mesenchymal precursor cells differentiate into chondrocytes and osteoblasts, culminating in the development of ectopic bone [55].

In the process of HO remodeling, connective tissue and skeletal muscle commonly exhibit infiltration of lymphocytes, macrophages, and monocytes around blood vessels. These lymphocytes proliferate extensively within and around muscle fibers, leading to significant muscle tissue damage [56]. As this process progresses, the infiltrated areas show excessive proliferation of fibroblasts and a marked increase in the number of newly developed blood vessels. This creates a necessary microenvironment for the clustering of mesenchymal cells and the differentiation of chondrocytes, ultimately facilitating the development of mature bone tissue [57]. In the subsequent synthetic phase, the degenerated muscle tissue is gradually replaced by a proliferative cell population with fibroblast-like morphology [16]. During this process, a local hypoxic environment induced by the inflammatory response further promotes angiogenesis and the occurrence of neovascularization [11].

**Figure 1:**
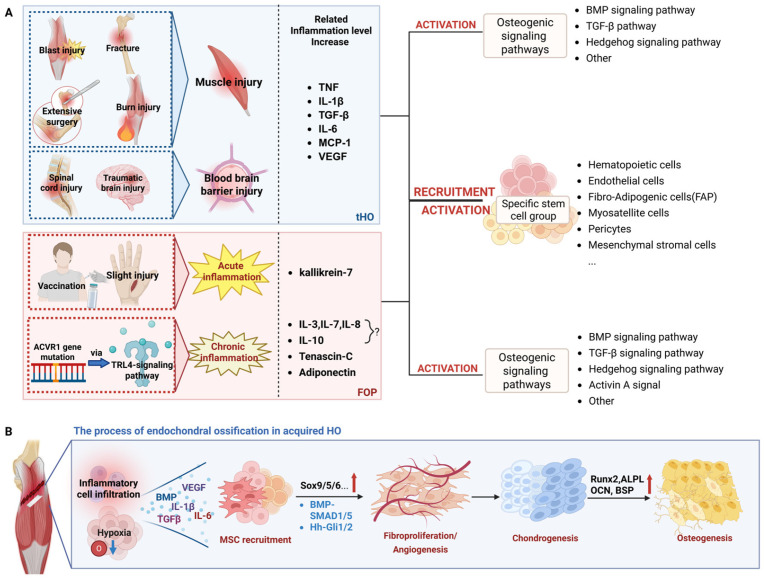
Mechanisms of traumatic HO (tHO) and Fibrodysplasia Ossificans Progressiva (FOP) (**A**). Pathogenesis of HO. tHO can be triggered by muscle injury and blood–brain barrier damage, accompanied by elevated levels of associated inflammatory cytokines. In FOP, the disease is broadly categorized into acute inflammation-induced FOP and chronic inflammation, involving distinct cytokines and signaling pathways. Both tHO and FOP recruit and activate specific stem cell populations, leading to the activation of corresponding osteogenic signaling pathways. (**B**) Mechanisms of Endochondral Ossification in Acquired HO. During acquired HO, inflammatory cell infiltration and a hypoxic environment develop in the injured muscle area, accompanied by the release of key cytokines such as BMP, VEGF, and IL-1β. Subsequently, mesenchymal stem cells are mobilized to the injury site, initiating fibrosis and angiogenesis. This is followed by the initiation of chondrogenesis, ultimately leading to heterotopic ossification via osteogenesis. Created in BioRender. CHEN, S. H. (2026). https://BioRender.com/6r84ts5 (28 March 2026).

Osteoprogenitor cells aggregate to form cartilage precursor structures, which gradually differentiate into chondrocytes, generating a cartilage template. Over time, these cartilage templates mature and hypertrophy, eventually being replaced by endochondral ossification, giving rise to the generation of mature bone tissue. In FOP, the impairment of the chondrogenic process not only leads to the formation of heterotopic bone but is also frequently accompanied by defective growth plate maturation, premature osteoarthritis, and structural joint abnormalities [58]. A notable characteristic of FOP patients is a significant increase in serum levels of cartilage-derived retinoic acid-sensitive protein (CD-RAP), an important biomarker of the chondrogenic process [59]. In contrast, in POH, the ossification process is primarily completed through intramembranous ossification. In this process, mesenchymal cells differentiate directly into osteoblasts without passing through an intermediate cartilage stage, subsequently forming ossification centers [60]. During embryonic development, intramembranous ossification plays a role in the formation of the skull, mandible, and central portion of the clavicle [61]. Moreover, it contributes to the formation of pathological bone bridges following growth plate injuries [62] (Figure 1).

### 3.2. The Mechanism of Major Subtypes of HO

#### 3.2.1. Mechanisms of Traumatic HO (tHO)

tHO is a complicated pathological process with core mechanisms involving injury, inflammatory response, and activation of abnormal osteogenic signaling pathways. Current evidence indicates a close association between the pathogenesis of tHO and the high plasticity as well as dynamic destiny of both skeletal stem/progenitor cells (SSPCs) and differentiated skeletal cells [63]. Following injury, these cells can be mobilized to function as active progenitors, capable of differentiating into chondrocytes or osteoblasts [45].

Injury triggers the migration of inflammatory cells, including macrophages and monocytes, into the local microenvironment, significantly elevating the concentrations of pro-inflammatory cytokines, including TNF, IL-1β, TGF-β, IL-6, and MCP-1. This inflammatory response further promotes the mobilization and stimulation of specific stem cell populations [64,65,66] and locally activates osteogenic signaling pathways [67,68]. Subsequently, this series of sequential events culminates in bone and cartilage formation, and the heterotopic bone tissue deposition [69]. Muscle injury is recognized as a critical initiating factor in HO development. Severe trauma, including fractures, blast injuries, and surgical procedures, frequently results in substantial muscle injury. The extent of muscle damage correlates with increased osteogenic potential in stem cells and an increased likelihood of HO, though the exact organism underlying this association is yet to be fully understood. Additionally, nerve injury, including spinal cord injuries (SCI) and traumatic brain injuries, is an important contributor to HO development. Nerve damage not only directly induces neurogenic HO but also disrupts the blood–brain barrier, leading to the secretion of various cytokines and osteogenic factors (e.g., BMPs) into the systemic circulation. This process amplifies the post-traumatic inflammatory response, creating a microenvironment conducive to HO formation [70,71,72,73].

#### 3.2.2. Mechanisms of Fibrodysplasia Ossificans Progressiva (FOP)

FOP is an uncommon genetic condition attributed to variations in the ACVR1 gene, with ACVR1 R206H being a notable example. The pathophysiological underpinnings of FOP entail dysregulation within the BMP signaling pathway and aberrant activation of the innate immune system. Currently, the diagnosis of FOP relies on ACVR1 mutation analysis, rather than a single or combination of biomarkers [74]. Research has shown that the ACVR1 R206H mutation induces a persistent pro-inflammatory condition. This condition occurs independently of acute disease flare-ups and may be regulated through the Toll-like receptor 4 (TLR4) signaling cascade [75]. Supporting evidence includes elevated concentrations of both pro-inflammatory cytokines (e.g., IL-3, IL-7, and IL-8) and anti-inflammatory cytokines (e.g., IL-10) in the serum of FOP patients. These findings point towards a substantial contribution of chronic inflammation to the pathophysiology of FOP [75]. However, subsequent research has revealed that these cytokines do not correlate with the FOP genotype or the presence of disease flares, highlighting the need for further exploration of the inflammatory factors involved in the disease process [74]. Additionally, Wang and colleagues further elucidated the role of TLR4 signaling in FOP. They discovered that inflammatory stimuli could activate TLR expression in connective tissue progenitor cells derived from FOP patients. This activation amplifies BMP signaling through both ligand-dependent and ligand-independent pathways. Significantly, the evolutionarily conserved signaling intermediate in the Toll pathway serves as a crucial link that integrates damage signals from TLR4 with the dysregulated ACVR1 signaling. This integration represents a fundamental mechanism for the cell-autonomous crosstalk between the innate immune system and BMP signaling pathways [76].

At the molecular level, adiponectin and Tenascin-C are highly correlated with the FOP genotype. Adiponectin, a hormone specifically produced by adipocytes, is involved in bone metabolism modification and HO processes. Its levels are regulated by hypoxia and inflammatory conditions and are closely linked to the metabolic activity of connective tissue progenitor cells [66,77,78,79,80]. Hypoxia conditions and inflammatory adipocytes are critical in the early stages of HO development [81], indicating that adiponectin could act as a biomarker for the initiation of the FOP microenvironment, reflecting susceptibility to soft tissue damage and preclinical lesions [74]. Tenascin-C, an endogenous activator of the TLR4 pathway, is persistently regulated in FOP patients, further supporting the notion that TLR4-mediated chronic inflammation is a key pathological feature of FOP. Additionally, kallikrein-7 is associated with the acute flare-up state in FOP, indicating its role in triggering inflammation [74]. FKBP1A (also known as FKBP12) is a negative modulator of the BMP signaling pathway [82,83,84]. Under normal conditions, FKBP12 interacts with type I receptors, inhibiting the activation of SMAD proteins. However, BMP binding to the receptor reduces the affinity between FKBP12 and the type I receptor, thereby relieving the inhibition on SMAD activation [82]. Therefore, in FOP, the reduced binding of the mutant receptor ACVR1R206H with FKBP12 is a central driver in the development of HO [82,83,84].

#### 3.2.3. Mechanisms of Progressive Osseous Heteroplasia (POH)

HO associated with GNAS mutations is characterized by the abnormal maturation of MSCs or undifferentiated progenitor cells residing in the dermal or subcutaneous adipose layers. Research on human MSCs has shown that reduced levels of Gsα protein promote osteogenic differentiation while suppressing adipocyte formation [85,86]. Studies using murine POH models have demonstrated that enhanced Hedgehog (Hh) pathway activity is essential for modulating mesenchymal progenitor cells (MPCs) differentiation and osteogenesis [87,88]. Specifically, GNAS homozygous loss-of-function mutations induce decreased cyclic AMP and protein kinase A levels in these animals [88]. In addition, the reduction in PKA activity further results in the disinhibition of Hh signaling, thereby enhancing osteoblast maturation and the development of heterotopic bone [88]. Additionally, in mice, the impact on adipose-derived progenitor cells caused by the absence of the paternal GNAS allele leads to subcutaneous ossification [89]. Experimental disruption of Gαs signaling in both embryonic limb mesenchymal tissue and mature subcutaneous progenitor cells results in Hh pathway activation in POH models. This activation is necessary and sufficient to induce osteoblast differentiation, underscoring the critical role of Hh signaling in HO associated with GNAS mutations [90]. Unlike FOP, which is driven by dysregulated BMP signaling, the pathogenesis of POH highlights the unique position of the Hh pathway in hereditary HO [32]. Consequently, conventional anti-inflammatory therapies, which are often effective against inflammatory forms of ectopic ossification, may provide limited or no therapeutic benefit in POH [32]. This distinction underscores that POH, resulting from mutations in the GNAS gene, represents a distinct entity for which targeted therapies aimed at correcting the specific Hh pathway dysregulation could offer a precise treatment strategy.

## 4. Major Signaling Pathways and Regulatory Networks in HO

**Figure 2:**
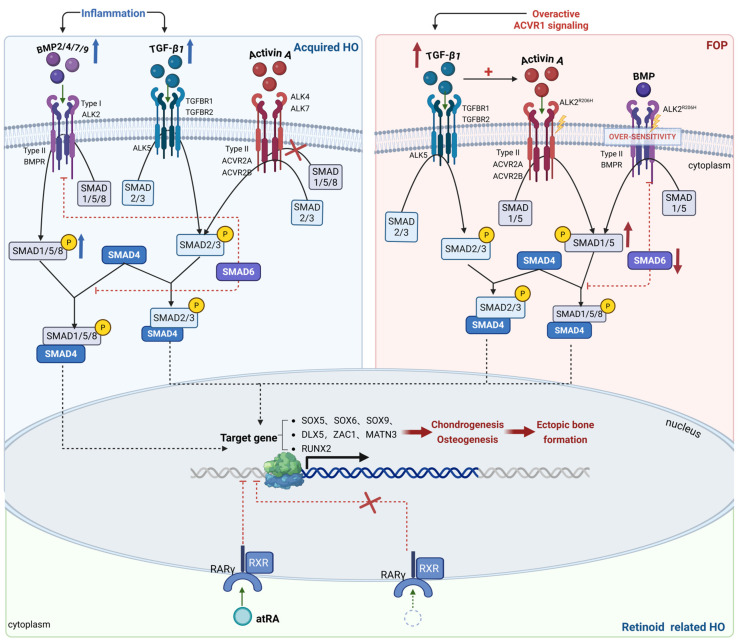
Acquired HO and FOP-related signaling pathways. In acquired HO, BMPs activate Smad1/5/8, while TGF-β activates Smad2/3 through the canonical pathway. In FOP, a mutation in the ACVR1 gene causes Activin A to activate and phosphorylate Smad1/5/8 via mutant ALK2, rather than Smad2/3. Phosphorylated Smad2/3 and Smad1/5/8 form a complex with Smad4, which then translocates to the nucleus to regulate target genes (e.g., Runx2) and trigger HO. In retinoid-associated HO, All-trans retinoic acid (atRA) typically binds to the retinoic acid receptor (RAR) to inhibit osteogenic differentiation; however, when atRA fails to bind RAR, this inhibitory effect is lifted, thereby promoting osteogenesis. Created in BioRender. CHEN, S. H. (2026) https://BioRender.com/12cxwtr (accessed on 13 March 2026).

Ectopic bone formation is driven by the dysregulation of multiple signaling pathways, which influence both the inflammatory phase and the recruitment of mesenchymal precursor cells [55]. These key pathways exert their effects through regulating a series of core genes: SOX9 acts alone or in coordination with SOX5 and SOX6 to participate in various stages of cartilage formation [91]; DLX5 primarily drives osteoblast differentiation [92]; ZAC1 influences embryonic skeletal development [93]; MATN3 promotes and maintains chondrogenesis [94]; and RUNX2 serves as a master transcriptional regulator of skeletal development, playing multiple critical roles in endochondral ossification and bone homeostasis maintenance [95]. This section will systematically elaborate on the regulatory network of these key signaling pathways in HO (Figure 2).

### 4.1. The BMP Signaling Pathway: A Central Driver of HO

Belonging to the transforming growth factor family, BMPs serve as vital signaling molecules for tissue homeostasis and differentiation, notably in cartilage and bone [7,96]. They transmit signals through plasma membrane-associated complexes comprising type I and type II receptors, both of which possess serine/threonine kinase activity [97,98]. Among the seven known type I activin receptor-like kinases (ALK1-7), BMP ligands show a binding preference for ALK1, ALK2, ALK3, and ALK6. And type II receptors include bone morphogenetic protein receptor type II (BMPRII), activin receptor type-2A (ACVR2A), and activin receptor type-2B (ACVR2B). The assembly of BMP receptors at the plasma membrane is a dynamic process. Critically, heteromeric complexes between type I and type II receptors are transient and unstable in the absence of ligand, in contrast to the stability of homomeric complexes. This kinetic distinction, together with competition between the two assembly states, serves as a key safeguard against inappropriate, ligand-independent signaling [99]. In the pathological context of FOP, the ACVR1-R206H mutant disrupts receptor complex dynamics and critically requires interaction with a type II receptor for full activation. Evidence showed that a constitutively active ACVR1 mutant (Q207D), mimicking FOP, depends on type II receptor binding for activation, even when the type II receptor’s kinase is inactive. This underscores the essential scaffolding function of type II receptors in the active signaling complex [100]. Upon ligand binding, the type II receptor mediates the trans-phosphorylation of the type I receptor, thereby activating downstream signaling pathways [96].

BMP signaling is divided into canonical SMAD-dependent and non-canonical SMAD-independent cascades. Within the canonical framework, activation of type I receptors induces phosphorylation of SMADs 1, 5, and 8 at their COOH-termini, thus activating them and promoting their interaction with the transcription factor SMAD4 [96]. This process involves phosphorylation of SMAD proteins by type II receptors such as BMPRII and ACTRIIA [101]. Also in these pathways, SMAD6 and SMAD7, both belonging to the inhibitory SMAD (I-SMAD) family, serve as negative regulators but through distinct mechanisms. SMAD6 primarily functions by binding to phosphorylated Smad1/5/8, thereby competing with Smad4 and preventing the formation of the active transcriptional complex [102]. By contrast, SMAD7 exerts its inhibitory effect by recruiting HECT-type E3 ubiquitin ligases, such as Smurf1 and Smurf2. In response to TGF-β signaling, SMAD7 binds to Smurfs in the nucleus and translocates to the cytoplasm, where it recruits these ubiquitin ligases to the activated type I receptor, ultimately targeting the receptor for proteasomal degradation and terminating the signal [103].

Conversely, the non-canonical SMAD pathways involve the activation of TAK1, p38 MAPK, ERK, or JNK, which initiate distinct signaling pathways [12]. This is initiated when BMP ligands bind to their receptors, leading to the recruitment and activation of the key upstream kinase, transforming growth factor-β-activated kinase 1 (TAK1) [104]. As a member of the MAPK kinase family, TAK1 acts as a central hub, which then phosphorylates and activates downstream MKKs, ultimately leading to the phosphorylation and activation of p38 MAPK, ERK1/2, and JNK [97,105]. The activated p38 MAPK, in turn, directly phosphorylates key osteogenic master transcription factors, including RUNX2, DLX-5, and OSX, thereby enhancing their transcriptional activity and stability—a critical step in driving osteogenic differentiation [105,106,107]. Importantly, both the Smad and the p38/ERK MAPK pathways are functionally cooperative and indispensable for BMP-induced osteoblast differentiation [108,109,110]. The phosphorylation of RUNX2 by MAPK is required for its efficient association with the Smad1/5/8-Smad4 complex, collectively maximizing the transcription of downstream osteogenic target genes [111]. Concurrently, BMP signaling activates the phosphatidylinositol 3-kinase (PI3K) pathway. This activation triggers the Akt and mammalian target of rapamycin (mTOR) signaling axis, which promotes cell survival and metabolic reprogramming to support the energetically demanding process of osteogenesis [112]. Furthermore, PI3K signaling can stabilize Smad1 by inhibiting glycogen synthase kinase 3 (GSK3), thereby potentiating the canonical BMP-Smad signaling output at the transcriptional level [112].

Extensively studied, the BMP signaling pathway is pivotal in regulating osteogenesis and chondrogenesis [113]. Aberrations in this molecular pathway are associated with several diseases, including FOP and neoplastic conditions [97]. Elevated levels of BMP2 and BMP4, which are significantly secreted following soft tissue damage, have been identified as key initiators of HO when introduced into mouse muscle tissue [55]. These factors are critical in supporting osteoblast differentiation and bone induction [114,115]. Additionally, the co-administration of BMP2 or BMP4 with other factors, such as VEGF and TGF-β, enhances endochondral ossification [73]. Furthermore, BMP2 has been associated with nerve inflammation, which may contribute to nervous system restoration and the mobilization or release of osteoprogenitor cells, further promoting osteogenic differentiation [52]. Notably, excessive BMP signaling can exacerbate the pro-inflammatory state of lymphocytes, potentially leading to manifestations of HO [17].

#### 4.1.1. Responsive Upregulation and Role of BMP Signaling in tHO

Reports indicate that BMP levels increase in response to inflammatory processes and are subsequently secreted into injured soft tissue. Following injury, there is a notable upregulation of mRNA levels for SMAD1 and SMAD5 [116]. Specifically, after brain injury, both BMP and its receptors, as well as their mRNA expression levels, are elevated [73,117,118]. Similarly, research on combat injuries has demonstrated that various osteogenic transcripts, including BMP-2, SMAD1, and ALPL, are significantly upregulated in cases with HO compared to those without HO [119].

In experimental models, the upregulation of BMP-related signaling further supports these findings. For example, in animal models of injury caused by trauma, transcription abundances of BMP-related genes such as BMP2/3, SMAD1, and ALK3 are elevated within wounded tissues [119]. Additionally, during HO formation induced by Achilles tendon transection, changes in the levels of TGF-β2, TGF-β3, and BMP mRNA are observed [120]. In a murine SCI model with induced HO, the expression levels of BMP2/4/7/9 are markedly elevated, and enhanced BMP signaling is noted in the quadriceps muscle post-SCI [121].

Exogenous activation of TGF-β/BMP pathways, including BMP2, BMP4, BMP9, or TGF-β, is widely utilized to induce ossification in vitro and in vivo. Notably, BMP2 and BMP9 show overexpression in human HO [122]. Furthermore, in models of burn and Achilles tendon transection, BMP-SMAD signaling markedly increases in the early stages of HO [123]. Collectively, these findings underscore the significant impact of the BMP signaling pathway on HO, providing a robust foundation for future research targeting therapeutic interventions.

#### 4.1.2. Responsive Upregulation and Role of BMP Signaling in FOP

Research has established that the BMP signaling pathway is critically involved in the pathophysiology of FOP. The fundamental cause lies in a gain-of-function mutation (predominantly the R206H substitution) in the ALK2 gene encoding ACVR1. This mutation leads to a loss of receptor autoinhibition, conformational changes, and a state of persistent hypersensitivity to BMP ligands, culminating in the overactivation of the BMP-SMAD1/5/8 pathway, which drives HO [55,124]. Studies reveal that in FOP, BMP signaling is disrupted, characterized by reduced BMP antagonist expression, increased phosphorylation of BMP signaling components, including BMP-specific SMAD proteins and p38MAPK, and aberrant BMP receptor trafficking. Specifically, the ACVR1-R206H mutant leads to aberrant activation of these non-canonical pathways. For instance, lymphocytes derived from FOP patients exhibit specific enhancement of p38 MAPK phosphorylation and activity upon BMP4 stimulation, accompanied by aberrantly high expression of its downstream target genes ID1 and ID3, an effect that can be blocked by a p38 inhibitor [125]. Animal models have also confirmed that inhibition of the PI3K/mTOR pathway reduces heterotopic ossification in FOP model mice [126,127,128,129]. These findings indicate that dysregulation of non-canonical pathways such as p38 MAPK and PI3K/mTOR is a key driver of the overall BMP signaling hyperactivation and ectopic bone formation in FOP.

It is worth noting that the manifestation of BMP transcriptional targets is elevated even when there are no external BMP ligands present [15]. Consequently, the mutant receptor remains overly sensitive to BMP ligands despite this autonomous loss of autoinhibition [15].

More importantly, the ACVR1 R206H mutation confers upon the receptor a disease-specific function: the aberrant response to another ligand—activin A. Activin A, an inflammatory cytokine secreted by immune cells, is a member of the TGF-β superfamily typically associated with SMAD2/3 signaling. Under physiological conditions, activin A signals through ALK4/7 and ACVR2A/B receptors, but does not activate the BMP-specific SMAD1/5/8 pathway via wild-type ACVR1 [130]. However, in FOP, the mutant receptor misidentifies activin A as a BMP-like agonist. Therefore, when tissue injury or inflammation elevates activin A levels, its binding to the mutant ACVR1 complex results in robust phosphorylation of SMAD1/5/8, thereby diverting this typical inflammatory cytokine into the osteogenic BMP signaling cascade [130,131]. This “ligand misrecognition” mechanism directly explains the clinical hallmark of FOP, where minor inflammation can trigger severe episodes of ossification. Therapeutic strategies targeting this axis have consequently become a primary focus in FOP drug development and have shown promise in clinical trials. The dysregulated BMP/activin A signaling is further amplified within the immune microenvironment of FOP. Research indicates that lymphocytes themselves are affected by the mutant ACVR1, and defects in their intrinsic regulatory circuits contribute to enhanced BMP signaling [132].

Within the immune system, T lymphocytes express diverse BMP Type I and Type II receptors, including the mutated ACVR1 variant [133,134], potentially leading to enhanced BMP signaling and a more intense local pro-inflammatory environment even without external BMP ligands. Additionally, other studies suggest that BMPs can trigger inflammation broadly. For instance, the use of recombinant human BMP2 to treat unicameral bone cysts resulted in an exaggerated inflammatory response [135]. Although it is currently recognized that HO diseases such as FOP are also closely related to BMP signaling, the exact mechanisms of abnormal activation of the signaling pathways therein remain to be determined [136]. But in any case, downregulating BMP expression to mitigate inflammation represents a promising strategy for managing HO.

### 4.2. The TGF-β1 Signaling Pathway: Regulator of Inflammation and Ossification

Transforming growth factor-β1 (TGF-β1) is a crucial signaling molecule in the bone microenvironment, particularly during bone formation and remodeling. Its levels are significantly elevated in response to bone injury, highlighting its key role in these processes [137]. Essential for tissue regeneration and wound repair, the TGF-β1 pathway modulates all stages: hemostasis, inflammatory response, cellular proliferation, maturation, and tissue remodeling [138]. It regulates a spectrum of processes, including cellular mitotic activity, lineage commitment, motility, tissue infiltration, and directional movement of fibroblasts and immunocytes, with roles varying by context and cell type [139,140].

In normal fracture healing, TGF-β is essential as it promotes the multiplication and differentiation of MPCs, stimulates extracellular matrix production, and attracts osteoblasts [5]. TGF-β is also pivotal in facilitating cartilage formation, callus development, and bone strength enhancement [141]. In vivo studies show that TGF-β accelerates fracture healing and improves bone remodeling. However, some research has indicated that elevated TGF-β expression levels post-trauma or surgical intervention are associated with abnormal ectopic bone formation, and this association is particularly evident around the injury or surgical site [138]. The TGF-β superfamily encompasses several members, including BMPs, TGF-β, activins, and inhibins. Within the canonical TGF-β/BMP signaling cascade, ligands attach to type I and type II receptors, activating SMADs as signal transducers. BMPs activate SMAD1/5/8, while TGF-β activates SMAD2/3. These activated SMADs subsequently form complexes with SMAD4, translocating into the nucleus to modulate the expression of target genes [142]. A key downstream target is the RUNX2 gene, a critical regulator of bone formation, which is notably overexpressed in tissues affected by ectopic ossification [143].

TGF-β-induced SMAD2/3 activation enhances osteoprogenitor migration and early differentiation while impairing later bone formation. Phosphorylation of SMAD2/3 causes the suppression of RUNX2 transcription, while SMAD3 recruits HDAC4 and HDAC5, further repressing RUNX2 function [143]. Conversely, bone formation is also modulated by the TGF-dependent non-SMAD pathway, where receptor engagement activates the TAB1-TAK1 complex, leading to p38 MAPK or ERK1/2 pathway stimulation. This activation enhances RUNX2 activity and supports osteoclast differentiation [107]. TGF-β serves as a dual modulator in bone metabolism, bridging the mechanisms of bone formation and resorption. It facilitates bone formation through mechanisms like RUNX2 phosphorylation, enrichment of osteoprogenitors, promotion of osteoblast proliferation, and apoptosis inhibition, while inhibiting bone resorption by reducing RUNX2 expression and function and impairing osteoclast maturation [144]. The increased activity of TGF-β has been confirmed in human HO tissues [145]. The pivotal involvement of the TGF-β signaling cascade in traumatic HO has been further elucidated. In particular, studies employing models of burn injury and tendon transection have demonstrated a marked upregulation of TGF-β signaling at the sites of HO formation. Specifically, TGF-β1 receptor engagement induces SMAD2/3 phosphorylation, facilitating their nuclear import and subsequent regulation of target genes involved in multiple cellular processes [138]. Moreover, in a traumatic HO model, the increased levels of p-SMAD3 in MPCs and widespread TGF-β signaling in macrophages post-injury indicate a notable upregulation of this pathway in both cell types prior to chondrogenesis initiation. Histological data support this finding, showing typical TGF-β activity in both macrophages and MPCs at the primary HO lesion, alongside significant alterations in TGF-β ligand and receptor expression within macrophages. These results emphasize the indispensable contribution of TGF-β signaling in trauma-associated HO, especially during the early cellular response and the stages prior to chondrogenesis [138].

In FOP, overactive ACVR1 signaling in macrophages leads to excessive TGF-β and proinflammatory cytokine secretion [75], and TGF-β has also been demonstrated to function as a critical FOP-specific activator of Activin A, as evidenced by the upregulation of both the INHBA gene and its corresponding protein expression [146]. The group Ruben D. de Ruiter has already shown that, in a model of osteogenic trans-differentiation, the chemical inhibition of TGF β-like signaling effectively prevented the differentiation of FOP fibroblasts even without the presence of recombinant ligands [147,148]. To quantify the level of Activin A production in FOP fibroblasts via ELISA, showing that TGF β1, as a disease-relevant recombinant cytokine, markedly enhanced Activin A expression in these cells, with notable increases observed at 24 and 48 h post-stimulation [146]. FOP-derived M2 anti-inflammatory macrophages exhibited a substantial upregulation of TGF-β expression, both following lipopolysaccharide stimulation and in the absence of stimulation [75]. Significantly, osteoclast-mediated resorption of calcified cartilage results in the release of abundant active TGF-β, which mobilizes mesenchymal stromal and progenitor cells within the HO niche to drive ectopic bone formation. Even during the maturation phase, active TGF-β continues to be released due to ongoing osteoclastic activity, thereby facilitating the advancement of HO [149]. Targeting TGF-β with neutralizing antibodies has achieved significant reductions in HO progression in both FOP and acquired HO experimental models [145].

### 4.3. The Activin a Signaling Pathway: A Pathogenic Mutation in FOP

FOP results from acquired mutations in the BMP receptor ACVR1 (ALK2), with the R206H variant representing the predominant mutation [150]. Although FOP and tHO share similar signaling pathways, they differ physiologically.

Activins, belonging to the TGF-β superfamily, are cytokines released by innate immune cells, such as neutrophils, macrophages, monocytes, and dendritic cells, following soft tissue injury [151,152]. Activin A typically activates signaling through type-I receptors (ALK4/7) and type-II receptors (ACVR2A, ACVR2B) [131]. These receptors, when activated, phosphorylate SMAD2/3, leading to downstream effects. Despite Activin A binding to wild-type ALK2, it fails to induce phosphorylation of SMAD1/5 or inhibit BMP signaling [153]. However, mutations in ALK2 enhance BMP ligand responses and sustain activity even without ligands. Recent studies reveal that Activin A, which normally activates SMAD2/3, can instead activate SMAD1/5 via mutant ALK2 [153]. Notably, an arginine-to-histidine substitution in ACVR1 causes aberrant upregulation of downstream signaling when Activin A attaches to the type II and type I receptor complex, leading to signals akin to those induced by BMP binding to SMAD1/5/8 [154]. In addition to acquiring abnormal responsiveness to Activin A, the ACVR1 R206H mutant also undergoes a fundamental shift in its regulatory function over canonical BMP ligands, such as BMP2/4. Under physiological conditions, wild-type ACVR1 acts as a homeostatic modulator within the BMP pathway by competing with BMPR1A or BMPR1B for their shared type II receptor, BMPR2. This competition suppresses BMP signaling and osteogenic differentiation mediated by BMPR1A/B [155]. In FOP, however, the ACVR1-R206H mutant completely loses this competitive inhibitory function. Studies have confirmed that, when co-expressed with BMPR1A or BMPR1B, the mutant instead synergistically enhances BMP signal output. This indicates that the functional dysregulation of ACVR1 R206H is twofold: on one hand, it gains the ability to misrecognize and respond to Activin A; on the other hand, its mode of interaction with other key type I receptors in the BMP pathway is reversed from normal “competitive inhibition” to “synergistic activation.” This functional transformation further disrupts the intracellular balance of BMP signaling and may constitute a crucial mechanism driving HO in FOP [155].

Research has shown that various cell lines with stable ALK2 R206H expression, including FOP patient-derived iPSCs, HEK293T cells, stem cells from human exfoliated deciduous teeth, and embryonic stem cells from a FOP mouse model, acquired the ability to activate SMAD1/5 signaling upon exposure to Activin A [54,124,156]. BMP typically signals through the type I receptor ALK2, whereas Activin A induces SMAD2/3 phosphorylation by binding to ALK4/7. Despite its ability to interact with ALK2, Activin A generally does not trigger SMAD1/5 phosphorylation and instead suppresses BMP signaling. In FOP, however, Activin A activates SMAD1/5 phosphorylation through the mutant ALK2, contributing to HO formation [153]. To model the cellular pathophysiology of FOP, iPSCs were derived from two affected individuals carrying the ACVR1 R206H mutation and two healthy controls. These cells were differentiated into iECs, generating paired lines (FOP-1, FOP-2, WT-1, WT-2) [130]. Subsequent transcriptomic analysis revealed that Activin A elevated pSMAD2 levels in both WT and FOP iECs, whereas SMAD1/5 phosphorylation was significantly enhanced only in FOP iECs. Additionally, Activin A elevated pSMAD1/5 levels in a manner dependent on both dose and time. Dysregulated Activin A signaling specifically altered the expression of SMAD1/5 target genes (ID1/2/3) in FOP-derived iECs [157]. To evaluate the involvement of type I receptors in Activin A/SMAD1/5 signaling, iECs were pre-incubated with K02288, an ACVR1/ACVRL1 inhibitor, or SB431542, an inhibitor of ACVR1B/C and TGFBR1. The results showed that only K02288 prevented SMAD1/5 phosphorylation, confirming that Activin A signaling occurs through ACVR1 [157].

Activin A levels rise rapidly during inflammation, with its effects regulated by interactions with natural inhibitors like follistatin and by diffusion gradients at the tissue level. The interaction of Activin A with type II receptors ACVR2A and ACVR2B and type I receptors ALK4 or ALK7 at the cellular level leads to SMAD2/3 phosphorylation. This signaling modulates target gene expression, influencing cellular responses [158,159]. Furthermore, Activin A, secreted by macrophages and other reactive immune cells during inflammation, triggers the secretion of pro-inflammatory cytokines, including IL-1β and TNF, along with mast cell recruitment, critical mechanisms in the initial stages of HO [69]. Evidence indicates that after tissue damage, macrophages, monocytes, and additional cell types localized at the HO site significantly increase cytokines like TNF, IL-1β, TGF-β, IL-6, and MCP-1 [51,160,161]. This cytokine upregulation drives the recruitment of specific stem cell populations [69] and stimulates pro-skeletogenic signaling pathways, ultimately leading to chondrogenesis, osteogenesis, and the development of ectopic bone.

Christina Mundy and her team demonstrated that systemic antibody administration effectively inhibited HO by approximately 70%, as measured through BV/TV ratios, irrespective of the induction method using rhBMP2 or rhBMP6 [69]. The introduction of the human mutant ACVR1(R206H) in murine models leads to FOP-like phenotypes, underscoring the pivotal role of ACVR1 dysregulation in FOP development [124,162]. Consequently, therapeutic strategies targeting ACVR1 or its downstream signaling pathways have become a focal point in FOP research.

### 4.4. Retinoic Acid Signaling: Inhibitory Regulation of Chondrogenesis

Retinoids, derived from the metabolism of vitamin A, include lipophilic compounds such as retinol, retinyl esters, and retinoic acid, playing crucial roles in embryonic growth and the maintenance of tissue and organ function [56]. The genomes of vertebrates encode three RAR genes (RARα, RARβ, RARγ) and three RXR genes (RXRα, RXRβ, RXRγ), each exhibiting distinct and dynamic expression patterns across various tissues and developmental stages [163]. Retinol, primarily inactive, is absorbed by cells via the STRAT6 transporter [164] and enzymatically transformed into the active metabolite all-trans retinoic acid (atRA) by cytoplasmic RDHs and RALDHs [165]. Although present in lower amounts, active retinoids like 9-cis and 13-cis RA are generated in specific tissue types [166]. Cellular retinoid-binding proteins contribute to the transportation of retinoids into the nucleus, enabling them to modulate the transcriptional activity of RARs and RXRs [167,168]. Research indicates that retinoids and RARs are essential for both prenatal skeletal formation and postnatal bone growth, influencing key processes like cell proliferation, differentiation, survival, and morphogenesis [167]. Regulating chondrogenesis is crucial for HO, as this pathological process depends on chondrogenesis. A key question is how condensed mesenchymal cells are directed toward chondrogenesis instead of alternative differentiation pathways like fibrogenesis or adipogenesis. Evidence shows this commitment is driven by pathways involving the SOX5/6/9 gene family and signaling pathways mediated by BMP and TGFβ superfamily members [169,170]. Studies indicate that the growth plate lacks endogenous active retinoids, suggesting RARs typically function as unliganded repressors to maintain growth plate activity. In HO, ectopic tissue masses first exhibit fibroproliferative characteristics before transitioning to chondrogenesis and endochondral ossification [56].

Evidence from studies utilizing RARE-LacZ reporter mice indicates that active retinoid signaling is lacking in limb mesenchymal condensations during chondrogenesis and cartilage development, but is highly active in adjacent progenitor cells, contributing to myogenesis and fibrogenesis [171]. Maurizio Pacifici’s studies reveal that suppressing retinoid signaling enhances chondrogenesis and growth plate activity, with unliganded RARs functioning as repressors. Studies using RARE-LacZ reporter mice [171] combined with biochemical analyses, confirm that early mesenchymal condensations, maturing cartilaginous tissues, and growth plates exhibit low levels of endogenous retinoids [168]. Additionally, Underhill’s group demonstrated that unliganded RARs are crucial for chondrogenesis, regulating critical chondrogenic genes and signaling molecules, such as Sox genes and BMPs, through detailed cellular and molecular research [172,173,174]. The authors observed that early pre-skeletal progenitors initially express RARα but switch to RARγ as they commit to chondrogenesis and differentiation. This transition is associated with significant reductions in RALDH2 and CRABP levels, alongside increased CYP26 expression [168]. Collectively, these studies affirm that chondrogenesis requires significant reduction or absence of active retinoid signaling [56] and gene transcription is initiated when ATRA binds to the RAR, while in its absence, gene expression is inhibited [163]. Therefore, if ectopic bone formation is to be promoted, it should be achieved by (i) removing endogenous retinoic acid and carrier proteins from early chondrogenic progenitor cells, (ii) inhibiting the anti-chondrogenic pathway via unlinked RARs, and (iii) promoting the expression and function of pro-chondrogenic pathways [34].

Comparative analyses of global gene expression (involving more than 360 genes) in limb bud progenitor cells demonstrated that those undergoing BMP-driven chondrogenesis displayed elevated levels of canonical BMP signaling components, such as phosphorylated SMAD1/5/8, and markers that promote cartilage formation or osteocyte differentiation, including DLX5, SOX9/5/6, ZAC1, and MATN3. In contrast, cells treated with all-trans retinoic acid (atRA) showed suppressed chondrogenic differentiation [56]. In contrast, atRA-induced inhibition of chondrogenesis reversed these pathways and restored active retinoid production via RALDH1A1, RALDHA2, RBP1, and CRABP2. Recent research clearly demonstrates that active retinoid signaling inhibits chondrogenesis by downregulating pro-chondrogenic pathways, such as BMP signaling, while upregulating anti-chondrogenic mechanisms [56]. This provides good ideas for preventing or treating HO.

### 4.5. The Hedgehog (Hh) Signaling Pathway: A Central Integrator in HO

The Hh signaling pathway represents a conserved system critical for embryogenesis, organ homeostasis, tissue repair, and regeneration following injury [175]. Disruptions to this pathway can cause impairments in the homeostasis of musculoskeletal tissues [176]. The Hh signaling cascade is orchestrated by Hh proteins, and its activation entails a complex, multi-step process. Among the key ligands within this pathway, Indian hedgehog (IHH) and Sonic hedgehog (SHH) are particularly crucial, as they are indispensable for the proper development of the musculoskeletal system [177].

Hh signaling activation occurs upon ligand binding to the transmembrane receptor Patched 1 (PTCH1). This binding event relieves the inhibitory influence that PTCH1 exerts on Smoothened (SMO), a cell surface protein that is specifically localized within the primary cilium. Primary cilia, projecting from the apical surface of mammalian cells, function as essential hubs for multiple signaling cascades [178,179]. Under normal conditions without Hh ligands, PTCH1 actively inhibits SMO function. Conversely, ligand binding to PTCH1 induces SMO activation. This activation leads to the degradation of suppressor of fused (Sufu), which in turn initiates downstream signaling through the GLI-Kruppel family of transcription factors, specifically Glioma-associated oncogene homolog 1 (Gli1), Gli2, and Gli3 [180]. Gli1 functions predominantly as a transcriptional activator, while Gli3 is predominantly a transcriptional repressor. Gli2, on the other hand, exhibits context-dependent functionality, capable of either activating or repressing transcription depending on the specific cellular milieu [180]. IHH is essential for osteoblast differentiation. Osteoblasts initially emerge near cartilage regions, especially close to hypertrophic and pre-hypertrophic chondrocytes. Within these chondrocytes, IHH signaling activates the transcription factor Gli2, which upregulates ALP and OCN activity. This demonstrates that IHH, via Gli2 activation, drives osteoblast differentiation. Thus, the elevated expression of IHH in hypertrophic chondrocytes is vital for this process [181].

Previous studies have shown that in mesenchymal and neuronal cells, Gαs signaling, as the physiological activator of PKA, inhibits Hh signaling through modulating PKA-induced Gli3 cleavage and generating cAMP [90,182]. Furthermore, a previous study indicates that the Yes-associated protein(YAP)-SHH positive feedback loop is a precipitating factor in the pathogenesis of HO in various murine models, such as POH, FOP, and traumatic HO [90]. In BMP-mediated injury-induced HO models, Hh and BMP pathways collaborate in a non-cell-autonomous fashion to establish a local microenvironment essential for HO formation [183].

The Hh signaling pathway is influenced by various regulatory factors. Research indicates that IHH is highly expressed in regions where ectopic cartilage forms, correlating positively with Gli1 levels [184]. Additionally, the expression of IHH is modulated by Runx2, a vital transcription factor involved in chondrocyte differentiation. In Runx2-deficient mice, chondrocyte maturation is disrupted, and IHH expression is significantly reduced [184]. Protein kinase A serves as an inhibitory modulator of the Hh signaling cascade, a pathway crucial for developmental processes [185]. Additionally, downstream Hh transcriptional regulation involves other signaling molecules, including casein kinase 1 (CK1), GSK3β, and β-Arrestin [180].

Additionally, Sufu shows negative regulation of the viability of the Hh signaling pathway, which restricts Gli protein activity by sequestering full-length Gli proteins within the cytoplasm. Loss of Sufu function brings about abnormal initiation of the Hh pathway, contributing to HO [186,187]. Although Hh signaling involvement in endochondral ossification-driven HO is well-established, the precise cell populations responsive to Hh signaling and the cell-autonomous mechanisms remain poorly characterized [188]. Furthermore, the deletion of FK506 binding protein 10 (Fkbp10) has proved to impact the function of the Hh signaling pathway, thereby exacerbating HO formation. Research has shown that deleting SMO in mice decreases ectopic cartilage formation, indicating Hh pathway suppression as a potential therapeutic approach for HO associated with Fkbp10 deficiency [184]. In summary, Hh signaling is integral to chondrogenesis and osteogenesis, and its aberrant activation or suppression has been linked to the pathogenesis of diverse skeletal pathologies, including neoplastic bone formation, degenerative joint disease, ectopic bone deposition, and other musculoskeletal abnormalities [188].

A review of previous studies highlights the pivotal involvement of the Hh signaling mechanism in the pathogenesis of both hereditary and non-hereditary HO.

Patients with POH exhibit a loss of functional activity in the Gαs protein. A null mutation in the GNAS gene disrupts Gαs’s ability to negatively regulate Hh signaling, leading to its dysregulation [189]. In POH patients, enhanced Hh signaling was observed at the sites of ectopic bone formation, as evidenced by analysis of tissue samples from these individuals [88]. Studies in GNAS-deletion mouse models have shown that the consequent upregulation of Hh signaling, accompanied by the onset of HO in the Achilles tendon, closely mimics the POH phenotype [180].

Supporting this mechanism, POH mouse models generated through the introduction of null mutations in the GNAS gene demonstrated that GNAS−/− mesenchymal cells exhibit increased secretion of SHH. This elevated SHH signaling was sufficient to induce osteoblast differentiation in adjacent wild-type cells. Furthermore, the loss of GNAS additionally triggered the transcriptional activation of YAP, leading to a direct enhancement in the expression of SHH. This elevated SHH secretion established a paracrine signaling loop, which drove ectopic osteogenesis by amplifying YAP activity, enhancing SHH production, and inducing osteoblast differentiation in nearby wild-type cells [90].

**Figure 3:**
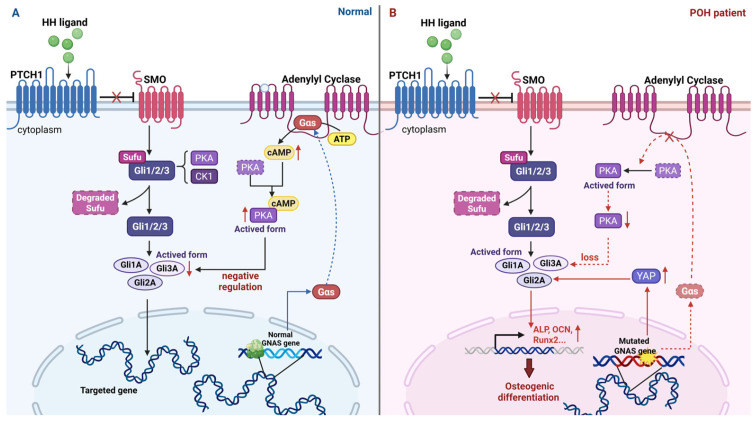
The pathological mechanisms of Progressive Osseous Heteroplasia (POH). (**A**) Normal activation state of the Hh signaling pathway and its associated molecules are shown. Normal GNAS genes can regulate Hh pathways by increasing cAMP levels and activating PKA. (**B**) In POH patients, GNAS gene mutations induce excessive Hh pathway activation, promoting osteogenic differentiation and ultimately resulting in HO. Created in BioRender. CHEN, S. H. (2026) https://BioRender.com/x35emns (accessed on 13 March 2026).

Experimental evidence further supports the utility of the Gαs-cAMP-PKA signaling axis in regulating HO through the suppression of the Hh pathway. In vitro studies using skeletal muscle progenitors and bone marrow stem cells demonstrated that GNAS deletion leads to reduced cAMP levels and PKA activity, accompanied by a significant upregulation of Hh target genes, including Ptch1, Gli1, and Hhip (all *p*-values < 0.001). The inactivation of PKA directly relieves the suppression of Hh signaling, enhancing the differentiation and mineralization of mesenchymal cells into osteoblasts [88]. Further mechanistic insights into POH have been gained from studies utilizing Prrx1-cre; Gnasfl/2 and Prrx1-cre; Gnasfl/fl mouse models. These investigations established that activation of the Hh signaling cascade is both essential and sufficient to trigger HO, while Gαs protein functions to suppress Hh signaling through the cAMP-PKA cascade [88]. Under normal conditions, Gαs tightly controls Hh signaling in soft tissues to avoid abnormal bone development [87]. These conclusions were reinforced by a Prrx1-Cremouse model with targeted GNAS deletion in limb mesenchymal progenitor cells, in which the mice developed severe progressive HO. The resulting mice exhibited severe progressive heterotopic ossification characterized by soft tissue syndactyly, joint fusion, tendon calcification, and skeletal abnormalities, closely resembling POH. Von Kossa staining and osteoblast marker analysis (Osterix, Osteocalcin) confirmed that ectopic bone formation occurred directly through intramembranous ossification, bypassing a cartilage intermediate stage. This underscores the importance of GNAS deletion in driving POH-like phenotypes via a cartilage-independent mechanism [88]. A murine model was employed to investigate the function of Gαs in intramembranous ossification during calvarial development, with a focus on the differentiation of calvarial chondrocytes. The study revealed that deletion of GNAS resulted in autonomous hypertrophy and ossification of mutated calvarial chondrocytes, which significantly enhanced HO within the cranial sutures and fontanelles. This pathological process was driven by the upregulation of Hh signaling in calvarial chondrocytes, predominantly through a ligand-free mechanism, which facilitated the transition of these cells toward an osteogenic fate [190] (Figure 3).

FOP, another hereditary HO disorder characterized by mutations in the ACVR1 gene, has not yet had its pathogenic mechanism fully elucidated in relation to the precise molecular mechanisms of Hh signaling, warranting further investigation [191]. Notably, IHH is essential for endochondral ossification, a process prominently observed in FOP [192]. In the FOP model mice with the ACVR1 (R206H) mutation, HO lesions exhibit upregulated expression of Ihh and its downstream targets, including Gli1 and Ptch1, while Shh expression is comparatively suppressed. Immunofluorescence analysis demonstrates that Ihh co-localizes with markers of chondrocytes (Sox9), osteoblasts (Osx, Runx2), as well as the transcriptional regulator Yap. These findings suggest that Hh signaling facilitates ectopic bone formation through the promotion of endochondral ossification.

Mechanistically, Yap/Tead4 cooperates with Smad1, an effector molecule downstream of BMP signaling, to directly bind the Ihh promoter region and activate its transcription, forming a positive feedback loop termed the ‘Yap–Ihh axis.’ [192]. Furthermore, Smad1 can directly interact with the truncated form of Gli3, highlighting functional crosstalk between these signaling pathways [193]. Smad4 specifically binds to GC-rich regions within the IHH promoter, supporting the notion that BMP signaling via Smad proteins induces IHH expression [194]. In vivo studies using Smad1/5 conditional knockout mice confirmed that BMP signaling directly regulates IHH expression in a Smad1/5-dependent manner. Ablation of Smad1/5 resulted in significantly reduced IHH levels, indicating that the BMP–Smad1/5 axis is essential for IHH transcriptional activation [195]. Additionally, secreted Ihh can activate Hh signaling in neighboring cells via paracrine mechanisms, further amplifying Yap activity and accelerating lesion expansion [192]. These findings underscore the central role of Hh signaling in FOP and provide a rationale for targeted interventions, such as Ihh-neutralizing antibodies.

**Figure 4:**
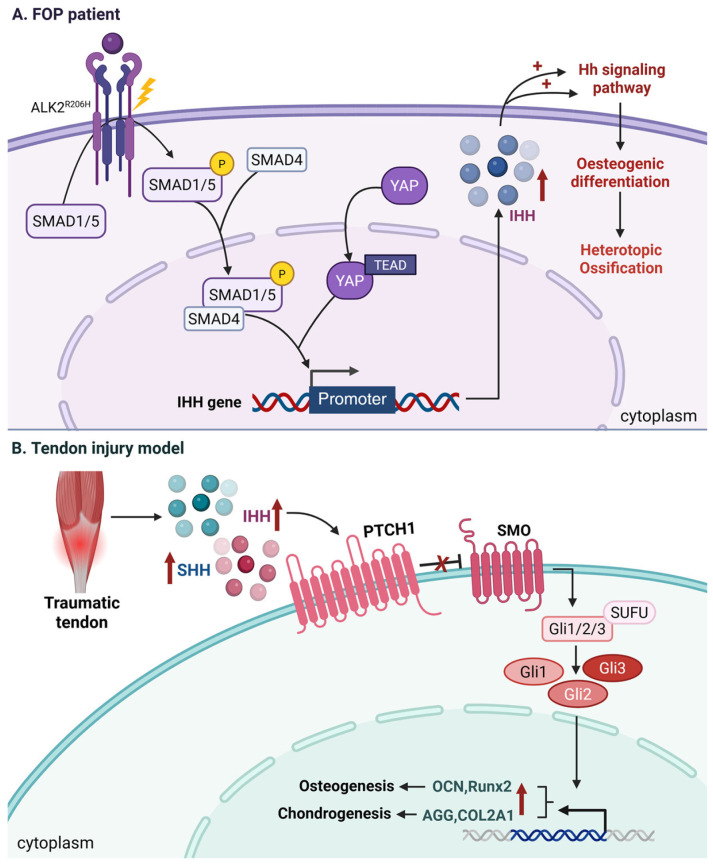
The pathological mechanisms of FOP and tHO in Hh signaling pathway. (**A**) ACVR1(R206H) mutation drives BMP/SMAD and YAP signaling to synergistically upregulate IHH expression, activating the Hh pathway and inducing osteogenesis. (**B**) Tendon injury upregulates IHH/SHH, which promotes both osteogenesis and chondrogenesis via the PTCH1/SMO/Gli axis, collectively leading to HO. Created in BioRender. CHEN, S. H. (2026) https://BioRender.com/kf5k8e1 (accessed on 3 April 2026).

In non-hereditary traumatic HO, MSCs undergo chondrogenic and osteogenic differentiation, a key process in its pathogenesis. Hh signaling directly controls MSC differentiation, facilitating the development of ectopic cartilage and bone in damaged tissues, thereby contributing to HO development [184]. Utilizing an Achilles tenotomy model, the pathological mechanisms underlying HO in tendons have been systematically elucidated. The findings demonstrate that tendon injury triggers significant upregulation of Hh signaling pathway ligands SHH and IHH. These ligands bind to the membrane receptor PTCH1, thereby relieving its inhibitory effect on SMO protein and subsequently activating the downstream transcription factor GLI1. The activated Hh pathway exerts direct regulatory effects on the differentiation fate of tendon-derived stem cells (TDSCs). It promotes osteogenic differentiation, as evidenced by increased expression of osteogenic markers OCN and RUNX2. Concurrently, the pathway induces chondrogenic differentiation, leading to elevated expression of cartilage-specific markers AGG and COL2A1 [177]. The precise molecular cascade connecting tendon trauma to SHH/IHH-mediated HO remains incompletely characterized and demands systematic investigation. Furthermore, Hh signaling modulates reactive oxygen species (ROS) activity in tendon-derived stem cells. Increased ROS activity drives the differentiation of osteogenic and chondrogenic cells, thereby promoting HO. Hh signaling also regulates antioxidant pathways in TDSCs, influencing ROS activity and contributing to tendon-derived HO [184] (Figure 4).

### 4.6. Another Signal Pathway

Hypoxia-inducible factors (HIFs) initiate the expression of genes that facilitate cellular adaptation to hypoxia [196,197]. They promote the formation of heme oxygenase [67] and regulate the coordinated growth of bone and vasculature during development [197]. Additionally, mTOR signaling pathways are pivotal in chondrogenesis, osteogenesis, and skeletal maturation [198,199]. Notably, the ACVR1 mutation associated with FOP has been demonstrated to enhance mTOR signaling [128]. In contrast, rapamycin effectively inhibits bone formation in FOP models [67,128], traumatic HO [67,200], and leptin-induced osteogenesis across in vitro and in vivo settings [201]. This suppressive effect occurs via the inhibition of mTOR complexes, including mTORC1 and mTORC2 [202].

## 5. Signaling Networks of Inflammatory Cytokines in HO Pathogenesis

In general, the initiation of HO is inseparable from the inflammatory cascade triggered by tissue injury [203]. All forms of HO appear to be closely associated with inflammatory responses, suggesting that a common pro-inflammatory pathway may be the key activator of bone formation in normally non-mineralized sites [75]. Following injury, the first immune cells recruited are T-helper cells and neutrophils, with monocytes/macrophages becoming the dominant infiltrating population approximately 24 h later [204,205]. These cells interact with resident fibroblasts, which play a pivotal role in all stages of HO: they initiate inflammation by releasing mediators such as IL-6, PGE2, GM-CSF, and CCL2 to recruit leukocytes, and convert acute inflammation into a chronic state, thereby creating a microenvironment conducive to fibrosis and aberrant repair [206].

Within the complex inflammatory network, a variety of immune cells secrete cytokine profiles that act directly on osteogenic progenitor cells, such as mesenchymal stem cells/progenitor cells. M1-type macrophages/monocytes are the primary cells responsible for producing key pro-inflammatory factors. The cytokines they secrete, notably IL-1β, IL-6, and TNF-α, are critical pro-inflammatory mediators whose levels are significantly upregulated during the early stages of HO [207]. IL-1β is not only a marker for M1 polarization and plays a crucial role in the initiation and progression of HO [208], but it is also a core product of NLRP3 inflammasome activation. In the inflammatory microenvironment of HO, TAK1, activated by cytokines, phosphorylates the IKK complex, leading to IκB degradation and the subsequent nuclear translocation of NF-κB. NF-κB initiates the transcription of the pro-IL-1β and NLRP3 genes [209]. Subsequently, the activated NLRP3 inflammasome induces caspase-1 activation, which processes pro-IL-1β into mature IL-1β for secretion [210,211]. Released IL-1β, by binding to the IL-1 receptor on the surface of bone progenitor cells such as TDSCs, not only acts as an inflammatory amplifier but can also significantly enhance the effects of other mediators like TNF-α, IL-6, and TGF-β1, synergistically promoting progenitor cell senescence and osteogenic differentiation [211]. Moreover, its accumulation is critical for inducing MSCs differentiation, thereby activating further ectopic bone formation [210,212]. TNF-α not only participates in early inflammatory response but also acts on various cell types to drive bone formation. Studies have confirmed that TNF-α can directly induce the phosphorylation of mTOR in fibroblasts and promote osteoblast differentiation and bone-like tissue formation by activating this signaling pathway [213] IL-6 is a key pro-inflammatory cytokine secreted by M1 macrophages. In the early stages of HO, its expression level increases in parallel with M1 macrophage infiltration, contributing to the formation of the initial inflammatory microenvironment [207]. Furthermore, oncostatin M (OSM), secreted by myeloid cells such as M1 macrophages, binds to its receptor complex (OSMR/gp130), widely expressed on the surface of cells, including mesenchymal stem cells. This binding activates the canonical JAK/STAT3 signaling pathway. Both in vivo and in vitro studies have demonstrated that this OSM/OSMR/JAK/STAT3 pathway directly promotes the osteogenic differentiation and maturation of bone marrow-derived mesenchymal stem cells [214,215,216,217,218]. In a mouse model of neurogenic heterotopic ossification (NHO) induced by spinal cord injury, OSM secreted by infiltrating macrophages accelerates NHO progression precisely through this pathway [219]. These findings establish OSM as a central mediator linking inflammation to abnormal osteogenesis. When fibroblasts are stimulated by injury, the production of chemokine CCL2 can play a key role in early inflammation by recruiting and polarizing macrophages to the injury site. This triggers a signaling cascade involving TGF-β/Smad, promoting collagen deposition and fibrosis, ultimately affecting bone progenitor cells and leading to abnormal bone formation [206].

Furthermore, following injury, neutrophils are recruited and polarized into an N2 phenotype under the influence of factors such as IL-8. These N2 neutrophils secrete stromal cell-derived factor 1α (SDF-1α), which activates the PI3K/Akt and β-catenin signaling pathways in bone marrow-derived mesenchymal stem cells via the SDF-1/CXCR4 axis, thereby driving MSC migration to the injury site—a critical step in initiating ossification [220]. The recruited MSCs, in turn, release factors such as IL-1α, IL-8, and TNF-α, which further recruit neutrophils and establish a positive feedback loop. Within this loop, SDF-1α released by neutrophils stimulates the osteogenic and chondrogenic differentiation of MSCs via the CXCR4-PI3K/Akt/β-catenin pathway [221,222]. On the other hand, neutrophils can also modulate key osteogenic markers such as BMP-2, TGF-β2, and RUNX2 in HO [37], and directly promote the differentiation of MSCs and progenitor cells into osteoblasts by activating BMP and TGF-β signaling [223].

Clinical studies indicate that elevated serum levels of various cytokines, including IL-3, IL-6, IL-7, IL-8, IL-10, MCP-1, and GM-CSF, in post-traumatic patients are associated with HO occurrence [51,161]. Blood samples from FOP patients also show significantly elevated levels of IL-3, IL-7, IL-8, IL-9, IL-10, IL-15, IL-17A, IL-1RA, GROα, eotaxin, and GM-CSF [75].

At the level of cellular signaling pathways, NF-κB and p38 MAPK pathways serve as core driving forces. In traumatic HO, significant upregulation of phosphorylated NF-κB p65 and phosphorylated IκBα can be observed early after injury in infiltrating macrophages, indicating the activation of the NF-κB signaling pathway [224]. Mechanistic studies have shown that this overactivation of the NF-κB pathway in macrophages promotes the expression and secretion of key osteogenic factors such as BMP6 and TGFβ1. These factors, in turn, act on surrounding osteoprogenitor cells, enhancing the BMP signaling pathway within them, thereby directly driving the osteogenic differentiation program and promoting ectopic bone formation [224].

In genetic HO such as FOP, this activation is more pronounced and persistent. The mutated receptor ACVR1 on macrophages selectively enhances the activity of both NF-κB and p38 MAPK, thereby exacerbating inflammation [75,225]. Peripheral blood monocytes from FOP patients exhibit heightened responsiveness to the TLR4 ligand LPS and show sustained NF-κB activation, demonstrating that NF-κB activation underpins inflammation in FOP [226]. Mechanistically, ACVR1 and TLR4 can regulate the expression of TAK1, a key node responsible for activating downstream NF-κB and MAPK pathways [227,228]. This hyperresponsiveness of the TLR4/TAK1 pathway, likely induced by endogenous DAMPs (such as HMGB1, HSP, hyaluronan) or PAMPs released after injury, explains the excessive inflammatory response during FOP flare-ups [229].

**Figure 5:**
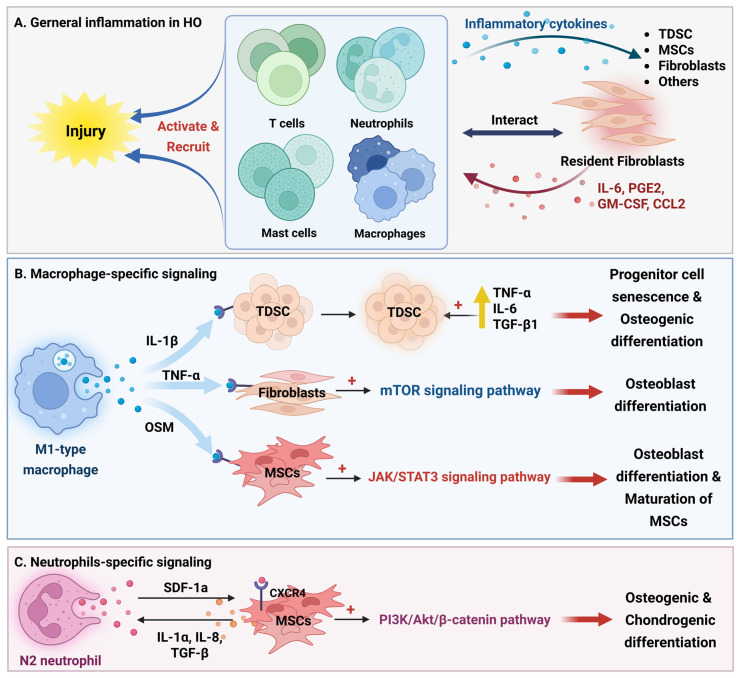
Schematic Overview of Immune Cell-Driven Osteogenesis in HO. (**A**) Tissue injury activates and recruits a diverse array of immune cells. This process leads to the release of pro-inflammatory cytokines, which interact with resident fibroblasts, collectively triggering the onset of the inflammatory-osteogenic cascade. (**B**) M1-type macrophages secrete key mediators, including IL-1β, TNF-α, and OSM. These factors activate distinct signaling pathways in target progenitor cells, promoting cellular senescence and osteogenic differentiation. (**C**) N2-polarized neutrophils contribute critically by secreting SDF-1α. SDF-1α binds to the CXCR4 receptor on MSCs, activating the downstream PI3K/Akt and β-catenin signaling axes. This activation drives both the osteogenic and chondrogenic differentiation programs of MSCs. Concurrently, MSCs release factors to establish a paracrine positive feedback loop. This loop further recruits and activates neutrophils, thereby amplifying their osteo-regulatory function within the developing heterotopic lesion. Created in BioRender. CHEN, S. H. (2026) https://BioRender.com/3z767tq (accessed on 3 April 2026).

In summary, the inflammatory response in HO is driven by a complex network of cytokines and their downstream signaling pathways. Pathways such as NF-κB, p38 MAPK, JAK/STAT, and the NLRP3 inflammasome/IL-1β axis not only mediate immune cell activation and the production of pro-inflammatory factors but also directly or indirectly prime mesenchymal progenitors and engage in crosstalk with osteo-inductive signals like BMP/TGF-β. The interplay among these pathways collectively shapes a microenvironment conducive to heterotopic bone formation (Figure 5).

## 6. Treatment Strategies for Heterotopic Ossification

To date, various treatment approaches for acquired HO have been employed, including anti-inflammatory drugs, bisphosphonates, localized radiotherapy, and surgical excision. Surgical removal of heterotopic bone remains a primary strategy for managing traumatic HO [230]. Nevertheless, the optimal timing and therapeutic outcomes of surgical intervention for HO have been subjects of debate, largely owing to the risk of recurrence post-surgery [42]. For hip arthroplasty, preoperative radiotherapy targeting the hip area, along with postoperative NSAID use, is a well-documented approach to prevent HO [33]. NSAIDs function by inhibiting cyclooxygenase (COX), thereby reducing prostaglandin synthesis—a critical factor in bone formation [231]. Systematic reviews have indicated that both selective and non-selective NSAIDs significantly lower post-traumatic HO compared to placebo [232,233,234]. One study examined the preventive effectiveness of ibuprofen compared to indomethacin for treating HO. It was observed that both drugs achieved higher effectiveness in preventing HO after primary THA compared to a control group without preventive medication [235]. Nonetheless, because these two agents are associated with a higher incidence of gastrointestinal complications [12], they potentially cause treatment discontinuation and reduced adherence. Etidronate, a simple bisphosphonate, has been explored as a preventive measure for HO due to its ability to delay matrix mineralization [12] and also be deemed to induce osteoclast apoptosis and inhibit calcification with controversy [42]. Typically, bisphosphonates are preferred for advanced stages of HO, particularly after the initiation of ectopic bone formation [236]. They work by promoting apoptosis in osteoclasts, thereby reducing calcification [237]. Bisphosphonates, while effective in inhibiting bone resorption, do not interfere with the synthesis of bone matrix. Consequently, mineralization processes may be recommended following the cessation of treatment [12].

Current evidence indicates that the primary therapeutic role of radiotherapy in HO is prophylactic. It is recognized for its ability to reduce the expression of pro-inflammatory cytokines, such as TNF-α, thereby mitigating local inflammatory responses and suppressing ectopic bone formation [213]. The therapeutic mechanism is thought to involve radiation-induced DNA damage, which inhibits the osteochondral differentiation of resident progenitor cells, including FAPs and muscle progenitor cells [238]. Clinically, Low-dose local radiation therapy has proven effective in decreasing the occurrence of HO after surgical procedures [43,239]. This is supported by evidence highlighting the role of prophylactic radiotherapy following procedures such as acetabular fracture surgery [240,241]. Furthermore, its application in preventing HO after elbow trauma has demonstrated both safety and efficacy in certain studies [242]. Nevertheless, the efficacy of radiotherapy remains controversial. Some studies report that in patients with elbow fractures, radiotherapy not only failed to reduce the occurrence of HO but also increased the risk of nonunion. Research by Honore et al. further suggests that local radiotherapy was ineffective in preventing the recurrence of heterotopic ossification in patients with spinal cord injury and traumatic brain injury [243]. Consequently, while delayed radiotherapy might modestly control the progression of established lesions, its necessity and effectiveness as a routine prophylactic measure, especially at anatomical sites other than the hip, are not firmly established [244]. Furthermore, the clinical decision to employ radiotherapy must weigh its benefits against potential risks, which include the possibility of radiation-induced malignancies [245] and complications including impaired wound recovery, advancing soft-tissue fibrosis, failure of fracture consolidation, and suboptimal osseointegration of uncemented hip prostheses [246,247].

**Figure 6:**
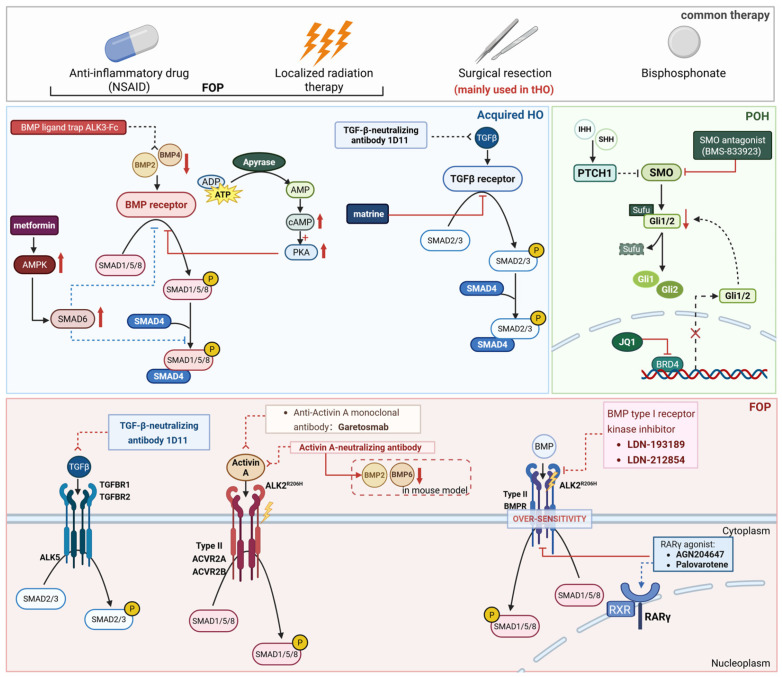
Relevant therapeutic strategies regarding the current prevention or treatment of HO. Clinical treatments for HO primarily include anti-inflammatory drugs, bisphosphonates, local radiotherapy, and surgical resection. In acquired HO, therapies target BMP receptor- and TGFβ receptor-related agents. For POH, treatment focuses on the SMO receptor. In FOP, interventions mainly target TGFβ, Activin A, RARγ receptors, and over-sensitive mutant receptors. Created in BioRender. CHEN, S. H. (2026) https://BioRender.com/85ecgny (accessed on 13 March 2026).

For mature HO following trauma, surgical resection is typically required, although achieving complete excision can be challenging, and recurrence is frequent [12]. In individuals diagnosed with FOP, HO exhibits an exceptionally aggressive course, characterized by widespread ectopic endochondral ossification beyond the skeletal system. This accumulation severely disrupts bodily functions and can result in premature death [248]. In such cases, HO is generally inoperable due to its reactive nature; surgical intervention may exacerbate and worsen HO. The onset of HO is frequently preceded by localized inflammatory processes [3]. Nevertheless, as our understanding of the underlying molecular mechanisms improves, targeted therapeutic strategies are beginning to be developed [12] (Figure 6).

### 6.1. Targeting the BMP Signaling Pathway

Due to the restricted effectiveness of existing treatments for preventing tHO, pharmacological approaches aimed at the modulation of BMP signaling have surfaced as a viable option for suppressing ectopic bone development [249]. These approaches encompass small-molecule inhibitors and recombinant protein-based ligand traps [250].

To explore factors in HO, samples from burn patients collected within 3 days of injury were analyzed and compared with those of unaffected controls. This analysis revealed an upregulation of pSMAD1/5/8 signaling and increased RUNX2 levels in the burn-induced samples, as well as a direct correlation between pSMAD1/5/8 signaling and adenosine receptor activity [101]. These findings imply that BMP signaling and ATP-mediated pathways might contribute to the development of burn-related HO. Similarly, MSCs from burn-injured adipose tissue showed elevated BMP signaling and ATP levels with enhanced osteogenic potential [3]. Apyrase, which hydrolyzes ATP and ADP to AMP, raises cAMP levels and inhibits SMAD1/5/8 phosphorylation [251]. In apyrase-treated mice, ATP cleavage led to higher adenosine and cAMP levels, reducing BMP signaling, HO, and improving limb mobility [101]. This therapeutic effect is attributed to the cAMP-dependent dephosphorylation of SMAD1/5/8, which subsequently inhibits HO and ameliorates joint contractures [101]. Experimental studies have indicated that metformin can inhibit BMP-induced SMAD1/5 phosphorylation by activating adenine monophosphate-activated protein kinase (AMPK). This leads to the upregulation of SMAD6, which subsequently suppresses the osteogenic differentiation of MC3T3-E1 cells [252]. The study indicated that metformin could prevent HO, likely by inhibiting the BMP signaling pathway. Given its affordability and safety, metformin holds significant potential for clinical application in the prevention and treatment of HO [252].

For FOP, characterized by activating mutations in ACVR1, recent treatments target excessive BMP signaling [3]. For instance, previous studies have demonstrated that LDN-193189, a BMP type I receptor kinase inhibitor with preferential activity against ALK2 and ALK3, effectively reduced HO and improved mobility in ACVR1 Q207D mice [253]. Additionally, LDN-212454, a more selective ALK2 inhibitor, demonstrated further advanced FOP treatment by significantly reducing HO in these mice. The BMP ligand trap ALK3-Fc, characterized by its strongest binding specificity for BMP-2 and BMP-4, markedly suppressed the osteogenic capacity of MPCs isolated from post-injury tissue in a burn/tenotomy model in vitro [3,249]. Amy L. Strong and colleagues found that LDN193189, LDN212854, and ALK3-Fc effectively inhibit traumatic HO. Unlike LDN193189, which caused severe neutropenia and decreased macrophage infiltration in a murine HO burn/tendonectomy model [254], ALK3-Fc showed good tolerance. In vitro analyses further showed that ALK3-Fc treatment resulted in decreased endochondral ossification, attenuated osteogenic differentiation, reduced proliferation, compromised regenerative ability, and altered transcriptional activity in MPCs [249]. The findings underscore the necessity for additional clinical research to evaluate the side effects and tolerability of BMP type I receptor kinase inhibitors and BMP ligand traps in the treatment of traumatic HO and FOP. TGF-β1 neutralizing antibody and ablation of TGF-β1.

Previous research has indicated that elevated concentrations of active TGF-β serve as both an inducer and promoter of heterotopic bone formation, suggesting that TGF-β may represent a viable intervention point for HO [145]. An initial analysis of HO specimens obtained from surgical and trauma patients revealed a substantial presence of pSMAD2/3-positive cells within the bone-like tissues. Their findings demonstrated that the administration of the TGF-β-neutralizing antibody 1D11 at various developmental stages (inflammation, chondrogenesis, and osteogenesis) significantly inhibited HO formation relative to the control group. Conversely, injection of 1D11 during the maturation phase (week 12) did not yield a statistically meaningful decrease in the incidence of HO. Furthermore, 1D11 administration significantly reduced the number of pSMAD2/3-positive cells and active TGF-β levels in the bone marrow of HO lesions [145]. In a mouse model of HO established following tendon injury, single-cell RNA sequencing and analysis of bone developmental pathways have been employed to elucidate the progression of HO [255]. Their study revealed dynamic functional interactions between TGF-β-secreting monocytes, macrophages, and chondrogenic progenitor cells, which were crucial to HO development. Notably, the conditional ablation of TGF-β1 in monocytes and macrophages resulted in a substantial reduction in chondrogenesis and HO formation [69].

D. Mao et al. first demonstrated the capability of matrine for HO treatment. Previous studies show that matrine can suppress SMAD2/3 phosphorylation to inactivate the TGF-β/SMAD signaling pathway, which is applied for the treatment of high glucose-incubated cardiac fibroblasts [256]. Based on an early study, they developed a murine model of traumatic HO via percutaneous puncture of the Achilles tendon. Their findings indicated that matrine suppressed the migration and osteogenic potential of MSCs in vitro conditions. In vivo experiments showed that marine significantly decreased the population of p-SMAD 2/3+ cells relative to controls and effectively attenuated HO progression in an ATP mouse model [5].

Moreover, TGF-β has begun to receive attention as a potential intervention focus for FOP. General delivery of the TGF-β1-neutralizing antibody 1D11 successfully suppressed HO development in a murine model of this invasive condition. These cumulative results indicate that targeting the TGF-β signaling pathway during the inflammatory, chondrogenic, or osteogenic stages effectively mitigates the development of HO [69].

### 6.2. Activin A Antibody for FOP Management

Recent studies indicate that the progression of HO in FOP necessitates Activin A-mediated activation of the mutant ACVR1 receptor [150]. Blocking Activin A has proven effective in averting HO in FOP mouse models, underscoring its pivotal role in driving this pathological process [136]. A compilation of various previously investigated therapeutic agents for HO includes Activin A antibody analogs, presumably including the following: (1) anti-Activin A monoclonal antibody [257], also called Activin A blocking antibody (Garetosmab), (2) Activin A-neutralizing antibody.

Developed via VelocImmune technology, Garetosmab is a humanized monoclonal antibody designed to specifically neutralize activin A, preventing its capacity to stimulate the mutant ACVR1 receptor associated with FOP [258]. In a humanized FOP murine model, Garetosmab effectively prevented the development of new HO lesions while simultaneously arresting the progression of existing lesions or promoting their regression [124,259]. The findings from the LUMINA-1 study, supported by earlier preclinical data from FOP mouse models, reinforce the significance of activin A as a critical ligand in HO in FOP [257,258]. These findings provide compelling evidence that Garetosmab is a potential disease-modifying treatment, effectively preventing HO while also diminishing the frequency and intensity of painful soft-tissue inflammatory episodes, thereby reducing the overall disease burden. Notably, Garetosmab demonstrated the most significant impact on preventing new HO lesions, suggesting its optimal intervention timing is likely in the early disease phase, before substantial functional deficits manifest in patients [258]. Further research on genetically accurate FOP mouse models (Acvr1 R206H FlEx and Acvr1tm1Glh) demonstrated that muscle injury-induced HO could be completely prevented with an activin A-blocking antibody [80,124].

Systemic administration of an Activin A-neutralizing antibody has been shown to effectively suppress the formation of subcutaneous HO [69]. The antibody also significantly reduced intramuscular HO in mice administered with rhBMP2 and rhBMP6, and histochemical analyses confirmed significant reductions in cartilage formation in neutralizing antibody-treated mice. At the same time, this successfully affected HO development as well as chondrocyte differentiation via exogenous activin A, further suggesting that strong expression of activin A in chondrogenic cells and chondrocytes is closely related to HO development [69].

Unlike FOP, traumatic HO (tHO) does not involve mutant ACVR1, and activin A has not been implicated in this form of HO [260]. The efficacy of a neutralizing activin A antibody (H4H10446P2) was evaluated in a tenotomy/burn model, where it was found that inhibiting activin A did not reduce tHO [260]. In contrast, anti-ACVR1 antibodies have been shown to inhibit tHO in wild-type mice. Notably, the specific monoclonal antibody mAb1, despite being administered upon induction, reduced but did not fully alleviate tHO in this model [261]. This confirms that while antibody-mediated inhibition of WT ACVR1 can impede HO, its effect is limited to conditions outside FOP [262].

In summary, targeting activin A proves effective in treating HO associated with FOP, primarily through its activation of mutant ACVR1. Nevertheless, this approach does not apply to other forms of HO, such as tHO. These differing responses highlight the need for tailored research and treatment approaches based on the specific pathology of each condition [260].

### 6.3. Hedgehog Signaling Pathway-Related Treatment

As a BRD4-targeting small molecule inhibitor, JQ1 effectively interferes with the transcriptional activity of Gli1 and Gli2, leading to the downregulation of Hh pathway target genes. Its capacity to block Hh signaling activation suggests its potential as a therapeutic agent for tendon and ligament HO [188]. It has been reported that Hh inhibitors, such as SMO antagonists and GLI antagonists, can treat various Hh-driven cancers [263]. The small molecule JQ1 has demonstrated efficacy in improving HO in the Ctsk-Cre Sufufl/fl mouse model, highlighting its therapeutic potential for HO. Previous studies have shown that Hh signaling is upregulated in human HO samples from the posterior longitudinal ligament, promoting chondrocyte differentiation in endochondral ossification [264]. Furthermore, it has been reported that Hh signaling contributes to chondroid HO in tendons within an injury-induced transgenic mouse model, with HO prevention achieved through the ablation of Hh signaling [183,265]. In both in vitro and vivo experiments, JQ1 effectively suppressed the accelerated chondrogenic differentiation of tendon-derived progenitor cells caused by upregulated Hh signaling. These findings suggest that JQ1 could be a prospective interventional option for endochondral HO in ligaments and tendons [188].

However, many Hh inhibitors, including SMO antagonists (such as BMS-833923) and SHH signaling inhibitors, have been primarily tested in isolated cells, with limited application in animal models. Therefore, the clinical relevance of these inhibitors remains an emerging area of research. For example, BMS-833923 significantly suppresses osteogenic differentiation, reduces ALP activity, and downregulates osteogenic gene expression in hMSCs. qRT-PCR analysis of BMS-833923-treated hMSCs showed reduced expression of Gli1 and PTCH1, confirming its specific suppression of the Hh signaling pathway [266]. As previously discussed, the Hh pathway is closely implicated in progressive osteotoposis pathogenesis. Therefore, Hh pathway inhibitors like JQ1 are expected to be novel therapies for the treatment of such conditions and provide a complement to traditional treatments.

### 6.4. RARγ Agonists: Suppressing Chondrogenesis

Acquired and congenital HO primarily involves endochondral processes, starting with mesenchymal condensation and chondrogenesis, followed by cartilage maturation and subsequent ossification [168]. It is well-established that chondrogenesis depends on transcriptional repression by unliganded nuclear RARs [173], and natural retinoid agonists like atRA have a beneficial effect on suppressing cell proliferation and differentiation [267]. When administered in high doses, it significantly inhibits bone growth and morphogenesis while simultaneously stimulating the bone resorption activity of osteoclasts [268]. Transcriptome analyses reveal that retinoid agonist treatment significantly downregulates genes involved in BMP signaling within chondrogenic cells [269].

Initial evidence suggests that retinoid agonists can effectively prevent and even reverse HO-like lesions [270]. Their experiment showed that mice treated with agonists experienced substantial reductions in ectopic cartilage, endochondral bone formation, bone marrow content, TRAP-positive osteoclasts, and vascular density, associated with decreased expression of SOX9, collagen X, collagen XI, and RUNX2 [168]. Retinoid signaling inhibits chondrogenesis and cartilage development in skeletal precursor cells by maintaining skeletal precursor cells in a prechondrogenic state and preventing their transformation into chondroblasts [270,271,272]. This insight positions retinoid signaling as a promising pharmacological target for treating FOP and other types of HO [273]. Pharmacological doses of RARγ agonists notably disrupt skeletal development and endochondral bone formation by diverting MSCs with chondrogenic potential towards alternative soft tissue differentiation pathways [163,274]. Although non-selective retinoids and RARα receptor agonists also inhibit HO, their efficacy is less pronounced [270].

AGN204647 has been confirmed as a potent RARγ agonist, which shows properties in HO in the studies. Intramuscular and subcutaneous injections of the RARγ agonist effectively inhibited HO. In transgenic mice with the ALK2Q207D mutation, AGN204647 effectively blocked ectopic bone formation, supporting its potential use in FOP [163]. RARγ agonist treatment reduced phosphorylated SMAD1/5/8 levels, crucial for transmitting pro-chondrogenic BMP signaling, and facilitated their proteasome-mediated degradation, reflecting broad disruption of the BMP signaling pathway and chondrogenic differentiation [158].

Both in vivo and in vitro studies confirmed the efficacy of these agonists in suppressing the chondrogenic phase of HO, without significant rebound effects post-treatment discontinuation [3]. Several studies document the antagonistic relationship between retinoic acid (RA) and BMP signaling [275], indicating that RARγ suppresses canonical BMP receptor signaling by diminishing SMAD phosphorylation [276]. These suggest that biphasic modulation of regional retinoid signaling could be advantageous for muscle regeneration. Initially, reducing local retinoid signaling may lessen interference with BMP activity, promoting muscle progenitor cell proliferation. Conversely, elevated RA signaling supports muscle cell differentiation and may counteract BMP signaling in damaged muscles, potentially lowering the risk of HO in muscle tissue [275]. Thus, while retinoid signaling can regulate chondrogenesis and HO, the specific mechanisms by which RARγ affects BMP signaling and muscle repair warrant further investigation.

Another agent being approved for the reduction in HO formation in both adult and pediatric populations is the Palovarotene, a selective RARγ agonist [277]. Research has demonstrated that Palovarotene can inhibit both chondrogenesis and HO in mice. It works by binding to RARγ, which helps prevent HO in patients with fibrodysplasia of bone by blocking BMP signaling and the SMAD 1/5/8 pathway in Caenorhabditis elegans. By inhibiting canonical BMP signaling, chondrogenesis, and affecting Inhba-expressing cells (Inhibin beta A-expressing cells), Palovarotene ultimately impedes chondrogenesis and stops HO, thereby promoting the repair and regeneration of muscle tissue [278,279,280].

Two phase II studies (NCT02190747 [281]; NCT02279095 [277]) have evaluated the safety and efficacy of palovarotene for FOP treatment. The findings support further clinical assessment of its ability to reduce new bone formation. A phase III clinical trial (NCT03312634) investigated the clinical effectiveness and safety of palovarotene in both adult and pediatric FOP patients regarding new HO formation [21]. The results indicated that palovarotene has an overall good tolerability; however, there is a potential risk of pulmonary complications in a considerable proportion of young patients, along with potential risks of reduced bone density and spinal fractures. Therefore, a thorough evaluation of the pros and cons is essential when considering palovarotene use in raising children [21].

In a mouse model of FOP, it was found that the content of Activin A remained at a high level even 21 days after injury. Suggesting that there may be a detrimental feedback loop in FOP, where Activin-A promotes HO formation, and in turn, HO stimulates Activin-A expression in general inflammatory cells, potentially exacerbating systemic HO [150]. To mitigate the systemic effects that contribute to HO, early intervention proves to be more effective than initiating treatment during episodes [150]. Looking back at previous studies, Activin A has emerged as a therapeutic target of interest as well as a preventive target.

### 6.5. Gene Therapy: A Novel Approach for Hereditary HO

In addition to various inhibitors targeting BMP signaling pathways and other specific signaling pathways, gene therapy is also gradually being proposed to treat HO, especially hereditary FOP. A gene therapy strategy utilizing AAV9-mediated delivery has been developed to express a codon-optimized human ACVR1 gene, implementing allele-specific silencing of ACVR1 R206H with AAV-compatible artificial miRNA, and employing an approach that integrates both gene replacement and silencing [282]. In their experiments, they demonstrated that AAV gene therapy successfully eliminated abnormal Activin A signaling and impeded both chondrogenic and osteogenic differentiation processes in murine skeletal cells and in patient-derived iPSCs with the human mutant ACVR1 allele. In Acvr1 R206H knock-in mice, local or systemic treatment significantly trauma-induced endochondral ossification. However, the inflammatory and fibroproliferative reactions of the injured muscle remained unchanged. These studies demonstrate that introduction of AAV9-mediated gene therapy at birth and in the early stages of adulthood effectively inhibits traumatic and spontaneous HO without negatively affecting chondrogenesis, bone growth, or bone remodeling [282]. This provides a promising option for AAV-based gene therapy as a prevention of HO in FOP.

## 7. Conclusions and Perspectives

HO represents a multifaceted pathological condition characterized by abnormal bone deposition in soft tissues, significantly impairing the patient’s quality of life. An analysis of existing data reveals significant alterations in key molecules across essential signaling pathways in traumatic HO, FOP, and POH, alongside potential therapeutic agents. The interplay of various signaling pathways, particularly BMP signaling and its interaction with inflammatory mediators, plays a central role in HO pathogenesis. For instance, gain-of-function mutations in ACVR1, a key receptor in the BMP pathway, are strongly associated with FOP, underscoring the need for targeted therapies. Current studies suggest that modulating BMP and ATP-related pathways could lead to decreased incidence of HO and improved mobility in individuals affected. Additionally, HO initiation can stem from multiple factors, including consequences of bone and joint surgery and inflammatory responses, leading to ossification occurring via endochondral and intramembranous processes. The initial inflammatory stage in soft tissues involves bone marrow, the site of injury, and local inflammatory macrophages, whose activity spurs fibroblast proliferation and neovascularization, facilitating the differentiation of chondrocytes into mature bone tissue. It is recognized that intervening early in HO to prevent its progression is a more effective strategy for favorable outcomes than later-stage medical or surgical interventions. Thus, targeting significant alterations in signaling pathways and critical molecules early in HO to manage molecular crosstalk and the dual modulation of local retinoid signaling is pivotal for future investigations. Furthermore, developing selective inhibitors for the BMP pathway, exploring adenosine-related mechanisms, and investigating genetic therapy present promising therapeutic possibilities for both FOP and tHO. Moving forward, an in-depth understanding of changes in distinct signaling pathways and the expression of key factors at the initial stages of inflammation, connective tissue degradation, and bone formation in HO, triggered by diverse factors, should be meticulously studied. A comprehensive grasp of the roles and regulations of each pathological signaling and key factors in early-stage HO scenarios is essential for devising precise interventions to either halt or reverse the progression of HO.

## 8. Literature Search Strategy

A literature search was conducted in the PubMed database, with no restrictions on the start date, and was completed in August 2025. Search terms included core disease names (e.g., “heterotopic ossification,” “fibrodysplasia ossificans progressiva,” “traumatic heterotopic ossification,” “progressive osseous heteroplasia”) and related molecular pathways (e.g., “BMP signaling,” “TGF-β signaling,” “Activin A,” “Hedgehog signaling,” “retinoic acid receptor”). Search terms were combined using Boolean operators. The literature screening process was managed with EndNote. Studies, reviews, and case reports directly related to the pathogenesis or treatment of heterotopic ossification were included, while editorials, commentaries, and other indirectly related literature were excluded.

## Data Availability

No new data were created or analyzed in this study. Data sharing is not applicable to this article.

## Abbreviations

The following abbreviations are used in this manuscript:

AAV9	Adeno-associated virus 9
ALP	Alkaline phosphatase
ALPL	Alkaline phosphatase, liver/bone/kidney type
AMPK	Adenine monophosphate-activated protein kinase
atRA	all-trans retinoic acid
BMP	Bone Morphogenetic Protein
BRD4	Bromodomain-containing protein 4
BSP	Bone sialoprotein
BV/TV	Bone volume per total volume
cAMP	Cyclic adenosine monophosphate
CK1	Casein kinase 1
COL2A1	Collagen type II alpha 1 chain
COX	Cyclooxygenase
CRABP	Cellular retinoic acid-binding protein
CYP	Cytochrome P450 family
DLX5	Distal-less homeobox 5
ELISA	Enzyme-linked immunosorbent assay
ERK	Extracellular signal-regulated kinase
FAPs	Fibro-Adipogenic precursors
Fkbp10	FK506 binding protein 10
FOP	Fibrodysplasia Ossificans Progressiva
Gli1	Glioma-associated oncogene homolog 1
GNAS	Guanine nucleotide-binding-subunit
GS	Glycine-serine
GSK3	Glycogen synthase kinase 3
HDAC4/5	Histone deacetylase 4/5
HEK293T	Human embryonic kidney 293T
Hh	Hedgehog
HIFs	Hypoxia-inducible factors
hMSCs	Human mesenchymal stem cell
HO	Heterotopic ossification
iECs	Induced endothelial cells
IHH	Indian hedgehog
IL-1β	Interleukin-1 beta
IL-6	Interleukin-6
INHBA	Inhibin beta A
I-SMAD	Inhibitory SMAD
iPSCs	Induced pluripotent stem cells
JNK	c-Jun N-terminal kinase
MATN3	Matrilin-3
MCP-1	Monocyte chemoattractant protein-1
MPCs	Mesenchymal progenitor cells
MSCs	Mesenchymal stem cells
NSAIDs	Non-Steroidal Anti-Inflammatory Drug
NT-3	Neurotrophin-3
mTOR	Mammalian target of rapamycin
OCN	Osteocalcin
OPN/SSP1	Osteopontin
OSM	Oncostatin M
p38 MAPK	p38 mitogen-activated protein kinase
PI3K	Phosphatidylinositol 3-kinase
PKA	Protein kinase A
POH	Progressive Osseous Heteroplasia
PTCH1	Patched 1
RA	Retinoic acid
RALDHs	Retinaldehyde dehydrogenases
RAR	Retinoic acid receptor
RARE	RA response element
RBP1	Retinol-binding protein
RDHs	Retinol dehydrogenases
rhBMP	Recombinant human bone morphogenetic protein
ROS	Reactive oxygen species
RUNX2	Runt-related transcription factor 2
RXR	Retinoid X receptor
SCI	Spinal cord injuries
SDF-1α	Stromal cell-derived factor 1α
SHH	Sonic hedgehog
SMAD	Suppressor of mother against decapentaplegic
SMO	Smoothened
SOX9	SRY-box transcription factor 9
SP	Substance P
STRA6	Stimulated by retinoic acid 6
Sufu	Suppressor of Fused
TAB1	TAK1-binding protein 1
TAK1	TGF-β-activated kinase 1
TDSCs	Tendon-derived stem cells
TEAD4	TEA domain transcription factor 4
TGFBR1	Transforming growth factor Beta receptor
TGF-β	Transforming Growth Factor Beta
THA	Total hip arthroplasty
tHO	traumatic HO
TLR4	Toll-like receptor 4
TNF	Tumor necrosis factor
VEGF	Vascular endothelial growth factor
YAP	Yes-associated protein
ZAC1	Zinc finger protein regulating apoptosis and cell cycle 1
